# Transdermal Delivery of Therapeutic Compounds With Nanotechnological Approaches in Psoriasis

**DOI:** 10.3389/fbioe.2021.804415

**Published:** 2022-01-24

**Authors:** Ning Li, Yeping Qin, Dan Dai, Pengyu Wang, Mingfei Shi, Junwei Gao, Jinsheng Yang, Wei Xiao, Ping Song, Ruodan Xu

**Affiliations:** ^1^ Institute of Basic Theory for Chinese Medicine, China Academy of Chinese Medical Sciences, Beijing, China; ^2^ Guang’anmen Hospital, China Academy of Chinese Medical Sciences, Beijing, China; ^3^ State Key Laboratory of New-tech for Chinese Medicine Pharmaceutical Process, Jiangsu Kanion Pharmaceutical Co., Ltd, Lianyungang, China; ^4^ Interdisciplinary of Nanoscience Center (iNANO), Aarhus University, Aarhus, Denmark

**Keywords:** nanotechnology, transdermal, drug delivery, psoriasis, nanomaterials

## Abstract

Psoriasis is a chronic, immune-mediated skin disorder involving hyperproliferation of the keratinocytes in the epidermis. As complex as its pathophysiology, the optimal treatment for psoriasis remains unsatisfactorily addressed. Though systemic administration of biological agents has made an impressive stride in moderate-to-severe psoriasis, a considerable portion of psoriatic conditions were left unresolved, mainly due to adverse effects from systemic drug administration or insufficient drug delivery across a highly packed stratum corneum *via* topical therapies. Along with the advances in nanotechnologies, the incorporation of nanomaterials as topical drug carriers opens an obvious prospect for the development of antipsoriatic topicals. Hence, this review aims to distinguish the benefits and weaknesses of individual nanostructures when applied as topical antipsoriatics in preclinical psoriatic models. In view of specific features of each nanostructure, we propose that a proper combination of distinctive nanomaterials according to the physicochemical properties of loaded drugs and clinical features of psoriatic patients is becoming a promising option that potentially drives the translation of nanomaterials from bench to bedside with improved transdermal drug delivery and consequently therapeutic effects.

## 1 Introduction

Psoriasis is a common chronic inflammatory, relapsed, and refractory skin disease, affecting about 2%–3% of the global population (Greb et al., 2016). Typical lesions of psoriasis are sharply demarcated erythematous plaques covered with silvery scales, which can coalesce into large areas of skin. The specific cause of psoriasis is unclear, but a variety of factors, involving polygenetic background, infection, stress, and smoking, have been shown to play a role in the etiology of this condition (Kamiya et al., 2019). Histologically, psoriasis presents as locally affected skin with features of hyperproliferation of premature keratinocyte (KC), infiltration of immune cells, and tortuous and dilated vessels (Rendon and Schäkel, 2019). In clinics, it is growingly recognized that psoriasis is a systematic disorder, reinforcing metabolic diseases such as obesity, diabetes, and cardiovascular diseases (Jensen and Skov, 2016). Besides, more than 16% of patients reported a higher level of psychological distress, even with suicide tendency (Cohen et al., 2016). Due to the complexity of psoriasis, the exact pharmaceutical targets and the corresponding interventions remain currently unsatisfactorily addressed (Daudén et al., 2020). Depending on co-morbidities and the severity of this disease, four major approaches have been clinically employed for psoriasis, including more direct treatment using topical interventions and phototherapy, as well as systemic therapies with either oral medications or injected biological reagents targeting specific receptors involved in psoriatic inflammation (Lebwohl, 2018; Rendon and Schäkel, 2019). Compared with systematic drug administration by oral route, the topical route of therapeutics is advantageous because it refrains medications from the hepatic first pass metabolism, renal filtration, harsh gastrointestinal environment, and off-target effects due to non-specific reactions (Rapalli et al., 2020). Moreover, since psoriasis is often a lifelong condition requiring repetitive interventions, the ease and convenience of application, improvement in drug bioavailability and pharmacological responses, as well as minimization of toxicity make topical drug delivery systems superior to other procedures. Though topical treatment is generally regarded as the first-line option in mild to moderate localized psoriasis (Rapalli et al., 2020), the resistance to permeability of most therapeutic molecules represents a major challenge in fabricating topical antipsoriatic drugs. In this scenario, advanced pharmaceutical dosage forms are of prime importance to be engineered or renovated.

**FIGURE 1:**
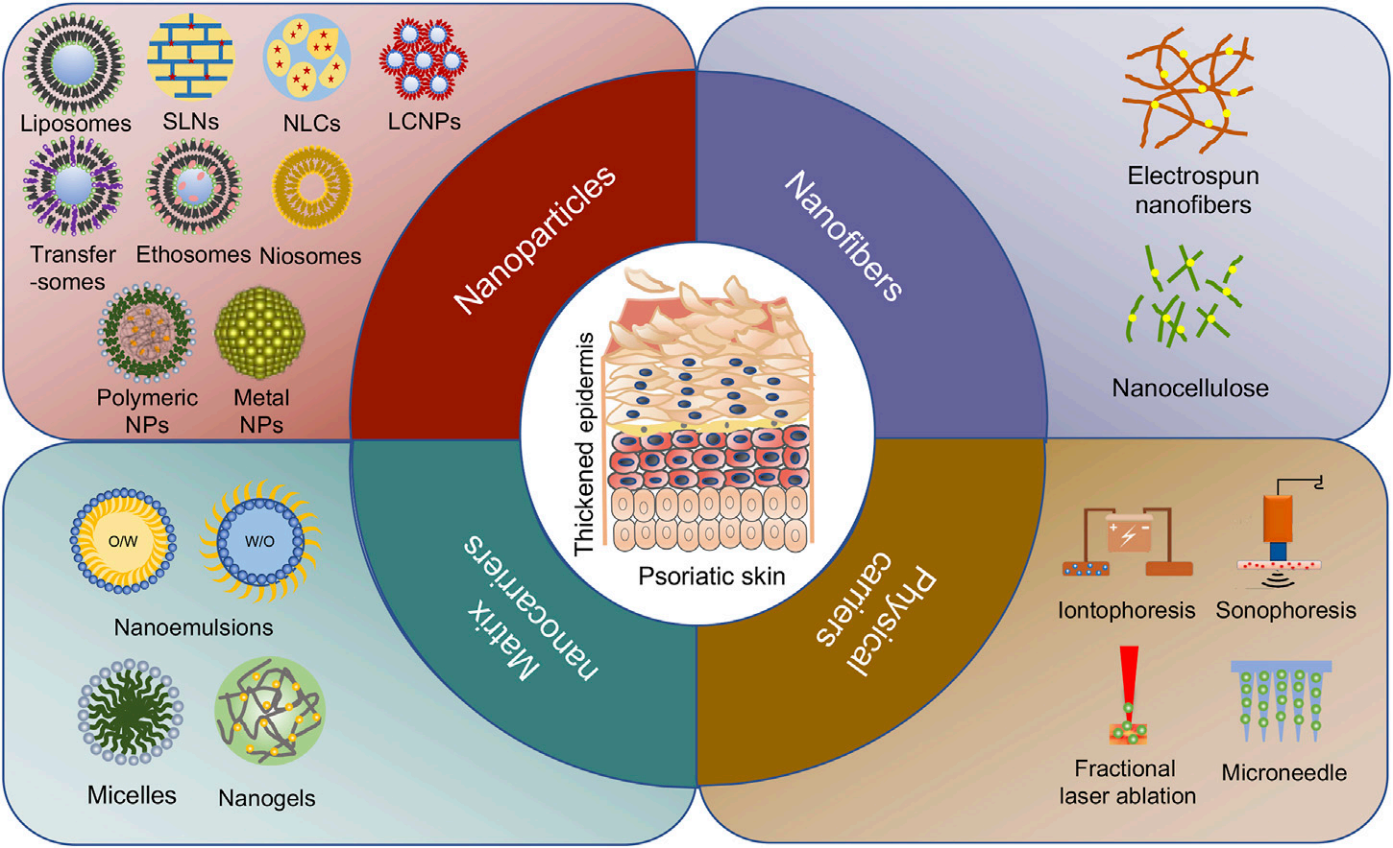
Different types of nanocarriers for psoriasis treatment. The passive diffusion of drugs *via* three pathways: intercellular, transcellular, and follicular routes are delivered by nanoparticles, nanofibers, and matrix nanocarriers. The active transfer of drugs is mainly transported by physical carriers including non-invasive delivery and invasive delivery.

Over the past two decades, we have witnessed the great success in medical nanotechnology, which has provided several opportunities and possible solutions to improve topical therapeutics for psoriasis (Saleem et al., 2020). In nanomedicine, materials within 1–100 nm are generally applied to design, fabricate, or modify therapeutic drugs. In the case of psoriasis, nanodrug-based topical therapies have demonstrated the following strengths over their conventional counterparts: (1) improving delivery capacities of insoluble drugs, thus maximizing drug bioavailability and efficacy (Lapteva et al., 2014a; Kang et al., 2018); (2) facilitating drug transport across skin barriers to enhance absorption and reduce drug dosage (Pinto et al., 2014; Mao et al., 2017; Freag et al., 2019); (3) controlling the release of drugs at precise dosages over a manageable period (Barradas et al., 2018; Langasco et al., 2018; Pukale et al., 2020); (4) protecting medications from degradation along with increased solubility (Soares et al., 2020; Lin Z. et al., 2021); and (5) providing targeting potentials when modified with cell-specific ligands, thus reducing adverse effects (Lapteva et al., 2014b; Shores et al., 2020; Lin Z. -C. et al., 2021). To date, three main possible routes are suggested for pharmaceuticals to penetrate skin barrier and reach the viable epidermis and dermis, including intercellular, intracellular, and transfollicular (Figure 1). Notably, most nanodrugs are merely validated at the pre-clinical stage, and more insightful characterization and evaluation are indeed demanded for their future translation from bench to bedside. Currently, with the exponential growth and diversity of nanotechnologies (Figure 1), such as nanoparticles (Table 1) and nanomatrix (Table 2) in addition to physical strategies (Table 3) for drug delivery, it becomes rather confusing in the rational selection of proper materials or technologies to fulfill an optimal topical treatment for psoriasis. Hence, this review desires to first distinguish the strengths and limitations of a variety of nanocarriers and methodologies that have been used in topical delivery drug systems in experimental psoriasis. Secondly, based on the features of different nanomaterials, we also intend to indicate the specific benefits of individual nanomaterials as clinical topical drugs for psoriasis.

**TABLE 1:** Topical applications of nanoparticles in drug delivery for psoriasis therapy.

Classification		Typical components	Carried drug	Size (nm)	Encapsulation efficiency (%)	Drug release (%)	Skin permeability	Stability (weeks)	*Experimental studies*	Transdermal delivery mechanism	Ref
				Zeta potential (mV)							
Lipid-based NPs	Liposomes	Phospholipids, cholesterol	Anthralin	116–199	≥97.2	26.3 ± 0.6	Permeate through the upper layers of the stratum corneum (SC)	N/A	Psoriasis patients	(1) Improve the hydration degree of the SC. (2) Change the structure of the epidermis by fusion with the SC and disrupt its lipid arrangement. (3) Permeate into the intercellular spaces *via* diffusion and capillary action	Fathalla et al. (2020)
				N/A							
			Capsaicin	368.5 ± 43	70.98 ± 2.36	24 h: 38.80 ± 2.57	24 h: ∼15% Permeate into the SC	N/A	Hairless rat skin		Gupta et al. (2016)
				N/A							
			Cyclosporine	111 ± 1.62	93 ± 2.12	120 h: 43.86 ± 4.85	N/A	4 (4°C)	IMQ rats		Walunj et al. (2020)
				41.12 ± 3.56							
			Curcumin	94–100	97	96 h: >80	Permeate into the dermis	1 (4°C)	IMQ rats		Yu F. et al. (2021)
				−22.0							
			Capsaicin siRNA	163 ± 9	92	N/A	Permeate into deep dermis	N/A	IMQ rats		Desai et al. (2013)
				35.14 ± 8.23							
			All-trans retinoic acid Betamethasone	70	>98	4 h: 100	24 h: 1%–5% (TRA), ∼8% (BT), Permeate into the dermis	N/A	HaCaT, IMQ mice		Wang W. et al. (2020)
				N/A							
	SLNs	Solid lipids	Methotrexate Etanercept	356 ± 2	88 ± 2	52 ± 4	8 h: 75%–80%, Permeate into the dermis	8 (RT)	Psoriatic skin	(1) Fusion with membrane. (2) Lipid-fluidizing property. (3) Occlusive effect. (4) Utilizing the skin transport pathways, including transcellular route, intercellular route and *trans*-appendageal route	Ferreira et al. (2017)
				−27 ± 4							
			8-methoxypsoralen	130.5 ± 1.2	99.6 ± 0.6	N/A	Permeate into the dermis, mainly in the SC	4 (RT)	Fibroblasts		Pitzanti et al. (2020)
				−35.6 ± 1.4							
				296.6 ± 49.5	N/A	∼70	Low permeation in hyperproliferative skin	N/A	Nude mice		Fang et al. (2008)
				−40.0 ± 5.9							
			Cyclosporine A	470.0 ± 4.6	92	21	Permeate into the epidermis-dermis layer	N/A	RAW 264.7 murine macrophage cell line		Trombino et al. (2020)
				N/A							
			Betamethasone (BD) Calcipotriol (CT)	188 ± 16	85.10 ± 2.02 (BD), 97.87 ± 0.08 (CT)	48 h: 45–56 (BD), 25–31 (CT)	4 h: Permeate into the dermis through appendageal pathway and intercellular route	12 (RT)	HaCaT, Mouse tail model		Sonawane et al. (2014)
				N/A							
			Cyclosporine A (CsA) Calcipotriol	90	70.93 ± 4.64	24 h: ∼50 (CsA), ∼80 (CT)	Non-detectable	N/A	HaCaT, IMQ mice		Arora et al. (2017)
				N/A							
	NLCs	Solid and liquid lipids	Methotrexate	221 ± 14	62.72 ± 0.94	N/A	24 h: 24.7 ± 2.3%, Permeate through the dermis	16 (RT)	Albino rats	(1) Occlusive effect of solid matrix. (2) Liquid lipids increase skin hydration	Tripathi et al. (2018)
				−33.6 ± 1.2							
				292 ± 9	172.7 ± 1.2–42.3 ± 2.0	2 h: 70, 8 h: 100	8 h: 5.8 ± 0.2% Permeate through the SC	4 (RT)	Pig ear skin		Pinto et al. (2014)
				−37 ± 3							
			8-methoxypsoralen	172.7 ± 1.2–42.3 ± 2.0	N/A	∼80	Low permeation in hyperproliferative skin	N/A	Nude mice		Fang et al. (2008)
			Tretinoin	79.5	92.31 ± 3.29	N/A	N/A	N/A	Mice tail model		Raza et al. (2013)
				−23.5							
			Fucoxanthin	416.3 ± 4.2	95.13 ± 0.76	N/A	N/A	N/A	HaCaT		Malgarim Cordenonsi et al. (2019)
				23.33 ± 1.36							
			Thymol	107.7 ± 3.8	89.1 ± 4.2	48 h: >70	24 h: Permeate through the dermis	11	BALB/c mice		Pivetta et al. (2018)
				−11.6 ± 2.9							
			Acitretin	223 ± 8.92	63.0 ± 1.54	30 h: 80.22 ± 3.40	N/A	N/A	Psoriasis patients		Agrawal et al. (2010)
				−26.4 ± 0.86							
			Cyclosporine A Calcipotriol	70.64 ± 5.28	79.05 ± 2.21	24 h: >80	Non-detectable	N/A	HaCaT, IMQ mice		Arora et al. (2017)
				N/A							
	LCNPs	Lipids, surfactants, water	Berberine oleate	137 ± 3.7	90 ± 1	1 h: 60	4 h: Permeate through Upper epidermis, 24 h: Permeate into Epidermis	N/A	IMQ mice	(1) Promote skin hydration. (2) Impart a sustained release behavior to the incorporated drugs	Freag et al. (2019)
				−38 ± 5.85							
			Tacrolimus	204.3 ± 1.78	99.3 ± 0.33	N/A	N/A	N/A	IMQ mice		Thapa and Yoo, (2014)
				N/A							
	Transfersomes	Phospholipids, edge activators, ethanol (<10%)	Methotrexate	∼100	−9 – 13 to −7 – −22	N/A	Permeate into the epidermis	13 (4°C)	Rat skin	(1) A highly deformable membrane that through skin pores easily. (2) Interact with lipid molecules in the polar headgroup region, resulting in an increase in the fluidity of SC	Zeb et al. (2016)
				−11.7 ± 1.2							
			Resveratrol	83–116	≥70	N/A	Not observed	8 (4°C)	HaCaT		Scognamiglio et al. (2013)
				−7–−22							
			Betamethasone dipropionate	242.8 ± 1.2	90.2 ± 0.48	0.75 h: 2.37–9.50	N/A	24 (4°C/RT)	Psoriasis patients		Gizaway et al. (2017b)
				−15.0 ± 1.0							
	Ethosomes	Phospholipids (0.5%–10%), ethanol (20%–45%)	Psoralen	120.77 ± 22.43	85.62 ± 0.76	N/A	Permeate through the dermis	N/A	Rat, Human embryonic skin fibroblasts	The flexibility and deformability of ethosomes facilitate drugs passing through SC and target deep skin layers	Zhang et al. (2014)
				N/A							
			Curcumin (Cur) Glycyrrhetinic acid (GA)	168.9 ± 15.6	N/A	N/A	Permeate through the dermis	N/A	BALB/c male mice		Guo et al. (2021)
				N/A							
			5-aminolevulinic acid	163.5 ± 0.9	10	N/A	Permeate through dermo-epithelial junction	N/A	Nude mice		Fang et al. (2009)
				−53.5 ± 0.9							
			Curcumin	∼200	∼90	24 h:>80	8 h: Permeate into the dermis	2 (4°C)	IMQ mice		Zhang et al. (2019)
				∼-30							
			Anthralin	201.5 ± 6.9	85.0 ± 0.6	18.3 ± 0.1	High permeation of the SC	N/A	Psoriasis patients		Fathalla et al. (2020)
				N/A							
			Methotrexate Salicylic acid	376.04 ± 3.47	91.77 ± 0.02	26.13 ± 1.61	8 h: 5.87 ± 0.01% Permeate through the SC	N/A	IMQ mice		Chandra et al. (2019)
				−20							
			Mangiferin	∼140	62–78	N/A	Permeate into SC	12 (4°C)	Fibroblasts, TPA mice models		Pleguezuelos-Villa et al. (2020)
				−38–−40							
	Niosomes	Non-ionic surfactants, cholesterol	Methotrexate nicotinamide	181.27 ± 1.44	71.05 ± 0.8	N/A	Permeate into the dermis, mainly in the SC	N/A	BALB/c mice	(1) The bilayer membrane made of non-ionic surfactant containing cholesterol has strong permeability. (2) High chemical stability	Yang et al. (2021)
				−24.53 ± 1.37							
			Ammonium glycyrrhizinate	79 ± 1.3	28.8	N/A	24 h: ∼25%, Permeate through the SC	12 (RT)	Healthy volunteers, CD-1 mice		Marianecci et al. (2012)
				−18.6 ± 1.5							
			Diacerein	477.8 ± 18	83.02 ± 4.9	7 h: ∼60	Permeate into the dermis, mainly in the epidermis	8 (4°C)	Albino rats		Moghddam et al. (2016)
				N/A							
			Acitretin	369.73 ± 45.45	90.32 ± 3.80	N/A	Permeate into the dermis, mainly in viable epidermis/dermis	12 (4°C)	HaCaT, Mouse tail model		Abu Hashim et al. (2018)
				−36.33 ± 1.80							
			Celastrol	147.4 ± 5.6	N/A	N/A	N/A	N/A	IMQ mice		Meng et al. (2019)
				−48.9 ± 1.1							
	Polymer-based NPs	Natural/Synthetic polymers	Curcumin	30.2 ± 7.97	78.45 ± 8.16	72 h: 49	24 h: Permeate into the SC, 48 h: A uniform fluorescent permeation in the SC, epidermis and dermis	N/A	IMQ mice	(1) The increase of interfacial area is beneficial to increase the interaction. (2) Controlled and sustained release of drugs by modifying the composition of polymer	Mao et al. (2017)
				16.7 ± 1.45							
			Spantide (SP) Ketoprofen (KP)	183	92.81 ± 2.17 (SP), 81.27 ± 2.26 (KP)	24 h: 20 (SP), 59 (KP)	Permeate into the dermis	12 (4°C)	Psoriatic plaque like model		Shah et al. (2012)
				5.34							
			Epigallocatechin-3-gallate (EGCG)	80–225	65	6 h: 50 24 h: 100	N/A	N/A	IMQ mice		Chamcheu et al. (2018)
				N/A							
	Metal-based NPs	Au, Ag	Methotrexate	4 ± 1	70–80	24 h: 95	Penetrate into the dermis	24 (4°C)	Keratinocytes	(1) Uptake by cells by active mechanisms, e.g., by endocytosis or by passive mechanisms, e.g., by diffusion. (2) Induce apoptosis, activate cytokines, and produce anti-inflammatory effects	Bessar et al. (2016)
				−32 ± 1							
			Fruit extracts of European black elderberry	20–80	N/A	N/A	N/A	4	HaCaT, Human psoriasis lesions		David et al. (2014)
				−20.9							

**TABLE 2:** Topical applications of nanomatrix in drug delivery for psoriasis therapy.

Classification		Typical components	Carried drug	Size (nm)	Encapsulation efficiency (%)	Drug release (%)	Skin permeability	Stability	*Experimental studies*	Transdermal delivery mechanism	Ref
Nanofibers	Electrospun fibers	High molecular polymer	Poly (propylene sulfide)	191 ± 39	N/A	N/A	N/A	N/A	Human dermal fibroblasts, RAW264.7 macrophages	Passive diffusion through skin appendageal routes by establishing a drug concentration gradient on the skin	Brooker et al. (2021)
			Salicylic acid, methyl salicylate, capsaicin	875 ± 49	100	N/A	N/A	Stable for 15 days, except methyl salicylate	HEK293-VR1, Healthy volunteers		Martínez-Ortega et al. (2019)
		Nanocellulose	Cellulose nanofiber	Curcumin	500	56.5 ± 9.7	N/A	Permeate through the dermis, mainly in epidermis	12 months (4°C)	IMQ mice	(1) Occlusion effect of the films. (2) Skin hydration effect of the films. (3) Passive diffusion through the appendageal routes by increasing the drug concentration gradient on both sides	Kang et al. (2018)
Other nanocarriers	Micelles	Surfactant, Macromolecule polymer	Tacrolimus	52.9	88.14 ± 0.20	1.5	Permeate into upper dermis	7 months (4°C)	Human skin	Preferentially deposited in skin wrinkles, between the corneocyte clusters, where there is a more permeable zone, which could increase drug delivery	Lapteva et al. (2014a)
			Silibinin	18.3 ± 2.1	75.8 ± 5.8	4 h: 21.8	48 h: 80.35 ± 3.37% Permeate through the full-thickness psoriatic skin	>3 months (RT)	IMQ mice		Chavoshy et al. (2020)
			Mycophenolic acid	20.75–25.08	N/A	24 h: 60, 48 h: 74	N/A	1 day (RT)	HaCaT cells (TNF-α-induced)		Supasena et al. (2020)
			ZnPC_4_	25	N/A	N/A	N/A	1 day	Psoriasis guinea pig’s model		Jin et al. (2015)
	Nano-emulsions	Oil phase, Surfactant and cosurfactant	Calcipotriol	170.8	89.2	36 h: 95.0 ± 4.0	N/A	>2 months (RT)	IMQ mice	(1) Increase the solubility and diffusivity of SC. (2) Extract and swell skin lipids to enhance penetration through the pores. (3) Permeate the scaly keratinized psoriatic skin through the hydrophilic pathways and pores between the skin cells	Wang Y. et al. (2020)
			Pioglitazone	182 ± 11.36	N/A	34 h: 73.6	Permeate through the SC	>60 days (RT/40°C)	AA mouse model, TPA mouse model, Healthy volunteers		Espinoza et al. (2019)
			Cyclosporine	159.9	N/A	3 h: 81.49	N/A	>3 months (4°C/RT)	Healthy volunteers		Musa et al. (2017)
			Rice bran oil	69 ± 17	N/A	N/A	N/A	>90 days (4°C/RT)	Psoriasis patients, Healthy volunteers		Bernardi et al. (2011)
			Clobitasol Propionate Calcipotriol	35.45 ± 2.68	N/A	36 h: 100	Non-detectable	N/A	SD rats, HaCaT, IMQ mice		Kaur et al. (2017)
			Curcumin Thymoquinone Resveratrol	76.20 ± 1.67	N/A	N/A	Permeate through dermis, mainly in epidermis	N/A	A-431 cells, IMQ mice		Khatoon et al. (2021)
			Clobitasol Propionate	240.5 ± 9.2	89.8 ± 7.11	24 h: 66.83 ± 2.05	N/A	6 months (4°C)	Rat UV-B dermatitis model		Dadwal et al. (2020b)
			Tacrolimus	93.37 ± 2.58	N/A	24 h: 80	Permeate into the SC and viable epidermis/dermis	N/A	A-431 cell, IMQ mice		Sahu et al. (2018)
	Nanogels	High molecular cellulose, high molecular polymer	Cyclosporine	361	N/A	24 h: 46.85	24 h: 46.85% Permeability through goatskin	N/A	TPA mouse model	Skin hydration	Li et al. (2020)
			Gemcitabine	200	48	12 h: 59	Penetrate the SC	N/A	Psoriasis mouse model, Human skin		Limón et al. (2019)
			Tacrolimus	170–1,000	68	12 h: 31	Penetrate the SC				
			Methotrexate	250–500	72	12 h: 41	Reach outer dermis layers				
			Curcumin	N/A	N/A	72 h: 30	N/A	N/A	IMQ mice		Mao et al. (2017)
			Methotrexate	196 ± 14	56.59 ± 5.7	5 days: 92	Penetrate into the dermis	N/A	IMQ mice, HaCaT, Porcine ear skin		Panonnummal and Sabitha, (2018)
			Clobetasol	132 ± 14	89.4 ± 3	72 h: 58	Penetrate through the dermis	<3 months (4°C)	IMQ mice		Panonnummal et al. (2017)
			Acitretin (Act) Aloe-emodin (AE)	138–238	∼94 (ACT), ∼89 (AE)	24 h: 60 (ACT), 68 (AE); 96 h: 96 (ACT), 98 (AE)	Mainly penetrate into the dermis	<3 months (4°C)	Mouse tail model		Divya et al. (2016)
												96 h: 96 (ACT), 98 (AE)
				Etanercept	155.16 ± 22.14	N/A	48 h: 19.7–34.9 (RT)	N/A	>2 weeks (RT)		Keratinocytes, Fibroblasts	Giulbudagian et al. (2018)

**TABLE 3:** Topical applications of physical strategies (active delivery) in drug delivery for psoriasis therapy.

Classification		Carried drug	Amount of drug	Application time	Skin permeability	Experimental studies	Transdermal delivery mechanism	Advantages	Limitations	Ref
Non-invasive delivery	Iontophoresis	Etanercept	1 mg/2.25 cm^2^	1 h	1 h: 80%, Permeate through epidermis and dermis	IMQ rat	Ca^2+^-mediated intracellular signal activation induced by IP, resulting in intercellular junction cleavage	As a drug reservoir, skin has the function of slow release and maintaining drug concentration	(1) Burn injury might happen. (2) Not available for high-molecular-weight compounds delivery	Fukuta et al. (2020)
		Methotrexate	45–60 mg	15 min	N/A	Psoriasis patients				Andanooru Chandrappa et al. (2020)
		Dexamethasone	8 mg	20 min	N/A	Nail psoriasis patients				Van Le and Howard, (2013)
		Hydrocortisone	0.5 mg	4 h	High dermis permeability	Human psoriatic skin				Dasht Bozorg et al. (2021)
		NF-KB decoy Oligodeoxynucleotide	10 μg/cm^2^	1 h	2 h: Permeate through the dermis	IMQ rat				Fukuta et al. (2021)
		Sonophoresis	Q-starch/miR-197 complexes	1.5–2.7 nmol	3 min (Ultrasound) and then 24 h (Q-starch/miR-197)	Permeate through the SC to the bottom layer of the epidermis	Psoriatic mouse model	(1) Barrier properties of the SC could be reduced. (2) Cavitation induces small pores on the skin surface and disorganization of the lipid bilayers within the SC	(1) Increase topical drug delivery. (2) Control the efficiency of transdermal drug delivery. (3) The reduction of skin barrier due to sonophoresis is reversible	(1) Ultrasonic administration is uneven. (2) The penetration of US to the skin varies from person to person. (3) The form of US radiation in psoriatic lesions needs to be explored	Lifshiz Zimon et al. (2018)
Invasive delivery	Fractional laser ablation	OS2966	0.5 mg	50–225 µs	Diffuse to the dermo-epithelial junction	N/A	The micro-thermal wounds produced by microbeams result in microchannels in skin	(1) Less thermal injuries. (2) Controllable and effective delivery of therapeutic agents. (3) Good compliance of patients	(1) Not applicable for patients with wide range of plaques. (2) *Trans*-epidermis water loss is significantly increased	Lapteva et al. (2019)
		Etanercept	N/A	50–225 µs	Diffuse into the dermis	N/A				Del Río-Sancho et al. (2020)
		Methotrexate	0.2 mg	175 µs	Permeability varies with laser setting	N/A				Nguyen and Banga, (2018)
		Methotrexate microemulsion	0.5 mg	N/A	N/A	Psoriasis patients				Ramez et al. (2018)
	Microneedles	Cas9 Ribonucleoprotein Dexamethasone	N/A	90 s	8 h: Diffuse into the dermis	IMQ mice	Directly creating micropores into SC	(1) Negligible pain and tissue damage. (2) Simple production. (3) Degradable materials	(1) May cause minor skin damage. (2) Mechanical properties of MNs are affected by the loading of drugs. (3) The modifiers that regulate the drug release rate of MNs also need to be further studied	Wan et al. (2021)
		Rapamycin	N/A	20 min	20 min: Diffuse into the dermis	N/A				Ramalheiro et al. (2020)
		Calcipotriol	N/A	N/A	Permeate through the dermis	IMQ mice				Liang et al. (2021)
		Pentaerythritol tetrakis (3,5-di-tert-butyl-4-hydroxyhydrocinnamate)	N/A	1 min	Limited to the SC	N/A				Gujjar et al. (2016)
		Cyclosporine A	1 mg/cm^2^	10 s	Dermis targeted delivery	N/A				Jeong et al. (2018)
		Tacrolimus (TAC) Diclofenac (DIC)	31.52 ± 1.78 μg/patch (TAC), 330.79 ± 9.66 μg/patch (DIC)	5 min	Permeate into the dermis and infiltrate into the joint cavity	IMQ mice, Arthritis rat model				Yu K. et al. (2021)
		siRNA	75 μg/patch	5 min	Permeate into the epidermis	N/A				Deng et al. (2016)
		Tofacitinib citrate	9 mg/patch	1–3 min	Diffusion of drugs in dermis is higher than that in epidermis	N/A				Cárcamo-Martínez et al. (2021)
		Methotrexate nanocrystals	2.48 mg/patch	30 s	Permeate through the dermis	N/A				Tekko et al. (2020)
			Methotrexate	65.3 ± 2.9 μg/patch	3 min	Permeate through dermo-epithelial junction				HaCaT, IMQ mice	Du et al. (2019)

## 2 Nanoparticles

Nanoparticles are nanosized particles with a structure that has at least one dimension less than 100 nm (Jeevanandam et al., 2018). The minuscule scale imparts nanoparticles large surface area-to-volume ratio, which endows them with great potentials for multifunctional surface modification. In general, nanoparticles themselves are not identified as simple molecules but as two or more materials assembled into three layers, including the surface layer, the shell layer, and the core (Khan et al., 2019). Owing to their exceptional characteristics, nanoparticles are capable of absorbing high quantities of molecules/drugs and circulating easily into organs and tissues, thus increasing their potential applications in biomedical investigations, such as chemical and biological sensing, bioimaging, and drug delivery (McNamara and Tofail, 2017). Typically, in drug delivery, which aims to localize, target, prolong, and has a protected drug interaction with the diseased tissues, the chemical composition of nanoparticles is considered as the critical determinant. In addition to composition, other elements such as size, shape, and surface characteristics may have additional effects on the physical, chemical, mechanical, and biological properties and behaviors of nanoparticles (Albanese et al., 2012).

Based on the chemical composition, nanoparticles are mainly classified into two categories: organic nanoparticles (e.g., liposomes and polymers) and inorganic nanoparticles (e.g., metals), both of which have been adopted for effective drug delivery in the treatment of psoriasis. Briefly, lipid-based nanoparticles that contain lipids are the most used in topical drug delivery because of their biocompatible, biodegradable, nontoxic, and nonirritating features, while polymer-based nanoparticles that consist of natural or synthetic polymers are preferred due to their intriguing features, such as versatility, sensitivity, and readily tunable chemical or physical modifications. As for metal-based nanoparticles (MNPs), such as gold and silver, their unique antimicrobial properties are considered one of the beneficial effects for the treatment of skin infections (Sánchez-López et al., 2020). Herein, different types of nanoparticles have been recruited for molecules/drugs to overcome skin barrier and subsequently improve the antipsoriatic effects of relevant molecules/drugs. The advantages and disadvantages of individual nanostructures are presented below to provide more experimental evidence and insightful understanding for a proper use of nanoparticles as antipsoriatic therapies.

### 2.1 Lipid-Based Nanoparticles

As an essential component of skin, lipids originated from sebum and keratinocytes play a vital role in maintaining skin intact, moisture, and health. Due to the similarity of lipid composition between lipid-based nanocarriers and epidermal lipids, the nanoparticles, such as liposomes, solid lipid nanoparticles (SLN), nanostructured lipid carriers (NLCs), liquid crystalline nanoparticles (LCNPs), transfersomes, ethosomes, and niosomes, have been introduced. In addition to providing a favorable biocompatibility, the greater penetration capacity of lipid-based nanocarriers into deeper layers of skin enabled penetration or permeation of medications through the stratum corneum (SC) (Shah et al., 2012). Meanwhile, the increased absorption of drugs by different skin layers potentially minimizes the drug dosage demanded and subsequent adverse drug reactions. Therefore, the topical application of antipsoriatic drugs in the form of lipid-based nanoparticles is considered to be the safest treatment for psoriasis based on current experimental and clinical evidence (Pradhan et al., 2013; Tripathi et al., 2018). However, due to the general low drug-loading capacity, insufficient permeability, and poor phase stability, application of lipid-based nanoparticles in psoriasis remains rather theoretical, requiring more sophisticated engineering strategies before being practically employed in clinics (Shah et al., 2012).

#### 2.1.1 Liposomes

Liposomes are spherical vesicular structures typically composed of phospholipids (natural and/or synthetic lipids) that are formed in aqueous solution, as initially described by Bangham et al. in 1965 (Bangham et al., 1965). Due to their amphiphilic nature (a hydrophilic head and two apolar hydrophobic chains) and bilayer structures, liposomes have been widely used as drug transporters for both hydrophilic (within the internal water phase) and lipophilic (within the lipid phase) molecules (Allen and Cullis, 2013). Among the three types of liposomes, namely, small unilamellar vesicles (SUVs, diameter 25–50 nm), large unilamellar vesicles (LUVs, diameter 50–500 nm), and multilamellar vesicles (MLVs, diameter 500–10,000 nm), according to the size and number of bilayer, LUVs are suggested to be the ideal structure for transdermal drug delivery in terms of drug-loading capacity, stability, and penetration efficiency across SC (Hope and Kitson, 1993). Over the years, a variety of methods for liposomal construction have been developed, including the thin-film method, injection method, extrusion method, and emulsification method (Wagner and Vorauer-Uhl, 2011; Alavi et al., 2017; Ajeeshkumar et al., 2021). Typically, in the preparation of LUVs, the extrusion method is mostly applied; however, due to the high loss of products caused by clogging in extrusion membranes, a large-scale application in industry is restricted (Ong et al., 2016).

Betamethasone dipropionate (BD) is a corticosteroid used in the treatment of psoriasis as anti-inflammatory reagents. However, the long-term use of BD often causes local skin atrophy and pigmentation. Considering that liposomes may assist in the transdermal delivery of drugs and thus reduce the dosage of BD and consequently the risks of skin atrophy and pigmentation, the liposomal formulation was constructed and evaluated. In a double-blind study, the beneficial outcomes of BD-loaded liposomes exhibited unexpectedly more in atopic dermatitis, as compared with psoriasis vulgaris, indicating that the conventional liposomes may not be a satisfactory drug carrier for psoriasis (Korting et al., 1990). The failure of BD-loaded liposomes in the treatment of psoriasis could be explained by either the unusual hyperkeratosis of psoriatic skin, which slows down the passage of BD liposomes, or the impaired skin integrity in atopic dermatitis, which accelerates the penetration of BD liposomes, suggesting that simple liposomal formulation may not be suitable for transdermal drug delivery in psoriasis.

Recently, Walunj et al. developed immunosuppressant drug Cyclosporine (Cyc)-loaded cationic liposomes and evaluated their antipsoriasis effects (Walunj et al., 2020). Dioleoyl-3-trimethylammonium propane (DOTAP), one of the most widely used cationic lipids, was utilized for the preparation of Cyc-loaded liposomes in order to solve the unfavorable physicochemical properties of Cyc, such as hydrosolubility and high molecular weight. Meanwhile, the positive charge on the surface of Cyc liposomes demonstrated a great affinity towards the anionic skin membrane, leading to improved efficaciousness in the imiquimod (IMQ)-induced psoriatic plaque model. Moreover, for better transdermal absorption, cell-penetrating peptides (CPPs) have been adopted and shown to increase cellular uptake and skin penetration of liposomes. In a more recent study, Yu and his colleagues designed CPP-modified liposomes for curcumin (Cur) as antipsoriatic therapeutics (Yu F. et al., 2021). By interacting with the C-terminus of Na^+^/K^+^-ATPase beta-subunit (ATP1B1), the CPPs were incorporated to electively open the paracellular pathway across cell epithelium, therefore assisting in skin permeation and the cutaneous retention of Cur-CPP liposomes compared to the Cur liposomes without CPPs. As a result, the Cur-CPP liposomes successfully improved visible symptoms such as erythema and silver scales, and decreased epidermal thickness as assessed by histology using the IMQ-induced psoriasis mouse model. Taken together, these findings proved that by continuously modifying the components of liposomes, the antipsoriatic effects of drug-loaded liposomes have evolved from being ineffective to effective.

The rapid and extensive evolution of liposomes has made this nanostructure one of the most safe and prominent vehicles for drug delivery. Until now, there are several liposomal formulations that have been approved by the US Food and Drug Administration (FDA) for a variety of diseases, such as cancer and fungal infections, a certain amount of which has reached the advanced phase of clinical trials (Bulbake et al., 2017). Some studies have reported that improved skin permeability could be achieved through various modifications of liposomes; nevertheless, no definitive evidence proved its effectiveness in psoriatic patients since it seems that a majority of drugs remain rather in the upper layers of the SC (Dayan and Touitou, 2000). Hence, the lack of transdermal delivery of drugs signifies that future efforts should be dedicated to improving the penetration of the liposomal system in psoriatic settings for clinical success. Accordingly, modifications in lipid phase by supplementing cholesterol, replacement of cholesterol with a suitable surfactant or penetration enhancer, and employment of a cationic or anionic surfactant have been utilized to enhance drug penetration and release into deeper layers of skin (Cevc and Blume, 2001).

#### 2.1.2 Solid Lipid Nanoparticles

As an alternative lipid particulate drug delivery system, SLN was first invented as small spherical particles by Gasco and Muller in 1991. Distinct from conventional liposomes, SLNs are particularly prepared by solid lipids that remain solid at room and body temperature, with the addition of suitable surfactants or emulsifiers to stabilize the lipid dispersion (Woo et al., 2014; Amoabediny et al., 2018). Solid lipids used in SLN preparation are glycerol behenate, stearic acid, Precirol^®^ ATO 5, Compritol^®^ 888 ATO, cetyl palmitate, etc. Based on the different physicochemical properties of lipids, drugs, and the applications of products, several preparation methods for SLNs have been established, such as hot homogenization, cold homogenization, and microemulsion techniques. As a more efficient topical drug delivery system (Jensen et al., 2011), the introduction of solid lipids contributes to the additional advantages of SLNs over conventional liposomes, as evidenced in drug loading and controlled release, protection of active compounds, and negligible skin irritation (Liu et al., 2020). In addition, the types of lipids and surfactants in SLN formulation affects SLN size, and the small size of SLNs with close contact to SC has been approved to greatly assist drug penetration through the skin barrier and consequently boost the delivery of drugs to a deeper depth of psoriatic skin. These beneficial effects of SLNs have been explained by the “occlusion effect”, which occurs due to the formation of an occlusive hydrophobic film on the top of SC when SLNs are administered topically, giving rise to a better hydration and transcutaneous penetration (Fang et al., 2008).

The use of topical corticosteroid BD in combination with the Vitamin D3 analog calcipotriol (CT) in the form of commercial ointment Daivobet™ is considered the gold standard therapy for mild-to-moderate psoriasis, whereas their adverse effects like skin atrophy and hyperpigmentation have been concerning. Sonawane et al. developed SLNs loaded with drug combination (BD-CT-loaded SLNs) by hot-melt extrusion connected with a high-shear homogenization technique (Sonawane et al., 2014). *In vitro* studies using rat and human skin demonstrated that BD-CT-loaded SLNs significantly increased the dermal absorption of BD/CT and delayed the abrupt growth of keratinocytes. Consistently, an *in vivo* mouse tail model demonstrated that administration of BD-CT-SLNs dramatically decreased the epidermal thickness without side effects compared with Daivobet™, confirming the antipsoriatic activity of BD-CT-loaded SLNs. Likewise, in the preparation of SLNs carrying the mixture of immunosuppressive drug methotrexate (MTX) and tumor-necrosis factor (TNF) inhibitor etanercept (ETA) by Ferreira et al., similar antipsoriatic effects were obtained when SLNs are applied as a topical solution (Ferreira et al., 2017). Collectively, these studies provide evidence that a significant improvement in the treatment of psoriasis could be achieved by SLNs.

Despite the fact that the production of SLNs is relatively simple, inexpensive, and scalable for industrial application, the involvement of solid lipid composition on the contrary gives rise to a perfect crystalline core in SLNs. Such a crystal lattice being highly ordered and compact has raised issues in drug-loading capacity during the drying process and drug leakage problems during the storage period. Therefore, some researchers have supplemented the original solid lipid matrix with a second solid lipid in an attempt to improve stability, enhance drug encapsulation efficiency, and prolong the release profile of loaded drugs in SLNs (Chantaburanan et al., 2017).

#### 2.1.3 Nanostructured Lipidic Carriers

To overcome the shortcomings of SLNs in drug solubility and drug leakage, NLCs, being regarded as the newest generation of SLNs, were designed by replacing up to 30% of the solid lipid mass of SLNs with liquid lipids. Though a certain amount of liquid lipids is supplemented, the produced NLCs are still in a solid form at room or body temperature when mixed (Haider et al., 2020). The commonly used liquid lipids for the preparation of NLCs are oleic acid, olive oil, soybean oil, sweet almond oil, squalene, and caraway essential oil, and the types of lipids together with the drug incorporated particularly determine the physiochemical properties and effectiveness of the end-products. When incorporating with liquid lipids, an imperfect and less ordered crystalline structure of NLCs was created, providing more space for drug dissolution and therefore affording NLCs with enhanced stability, augmented loading capacity, and minimized drug expulsion in contrast to SLNs. Apart from the above benefits, the nanosized NLCs optimized by factorial design also show a significant occlusion effect that is suitable for topical administration.

MTX, one of the most effective and widely used immunosuppressant drug in the treatment of extensive-to-severe psoriasis by oral or parenteral routes, was proposed for topical application because of the reported adverse effects after a long-term treatment, namely, bone marrow suppression, loss of appetite, hepatotoxicity, and cirrhosis. Unfortunately, topical use of MTX did not show significant clinical effects due to its low penetration across a highly packed SC of psoriasis. Agrawal et al. developed a gel-based, MTX-entrapped NLCs by the solvent diffusion technique, and evaluated its topical therapeutic responses in both *in vitro* and *in vivo* studies (Agrawal et al., 2020). MTX in the formulation of NLCs demonstrated a better antipsoriatic efficacy as shown by the reduced psoriatic area and severity index, together with ameliorated histopathological alterations in the skin and ears of IMQ-induced psoriatic animals. Meanwhile, MTX delivered by NLCs had increased SC deposition and a prolonged and sustained release of MTX. Moreover, it has been suggested that the combination of CT with MTX as a topical therapy has strengthened antipsoriatic effects; however, due to the extremely different polarities between CT (partition coefficient log *p* = 4.6) and MTX (log *p* = −2.2), the incorporation of these two drugs in a single vehicle is troublesome. Aiming to assess the potential of NLCs loaded with both lipophilic CT and hydrophilic MTX, Lin et al. optimized the methodology for the development of the NLCs using the high-shear homogenization technique (Lin et al., 2010). When skin permeation efficiency was evaluated in *in vitro* and *in vivo* studies, both CT and MTX showed increased feasibility of transporting across the skin barrier when carried within NLCs. Besides, since the two drugs incorporated in one formulation could be applied to the skin directly without waiting for a determined duration after the first drug vehicle is applied, a better patient compliance should be achieved.

Though both NLCs and SLNs have shown their potentials as delivery carriers for topical therapy and cosmeceutical applications, some comparative studies suggested that the optimized NLC systems containing antipsoriatic agents had better skin permeation and skin retention of drugs due to their better occlusive effect and reinforced tightness of junctions between drugs and SC (Fang et al., 2008; Agrawal et al., 2015; Arora et al., 2017). Therefore, compared to SLNs, an NLC-based delivery system is more advisable for psoriasis treatment.

#### 2.1.4 Liquid Crystalline Nanoparticles

LCNPs are formed by breaking down the bulk liquid crystalline, which is a matter between liquid and solid crystalline arrays, to nanosized particles composed of amphiphilic lipids with anisotropic structures, such as inverse hexagonal and cubic mesophases (Zeng et al., 2012; Silvestrini et al., 2020). Glycerol monooleate (monoolein, MO), a nontoxic, biodegradable amphiphilic lipid, is commonly used for the generation of LCNPs owing to its distinct property of polymorphism. The use of MO results in a highly ordered self-assembled structure with two discrete water channels separated by a lipid bilayer where different types of hydrophilic, lipophilic, or amphiphilic drugs could be localized based on their solubility properties (Freag et al., 2019). To date, LCNPs have attracted great interest as promising carriers in the treatment of skin diseases or cosmetology. This is mainly because of the bioadhesive characteristics of the biological membrane-like structure and the penetration-enhancing effect of involved materials, which are capable of modulating and disturbing the lipid phase in the SC barrier.

Berberine (BBR) is an isoquinoline alkaloid with a great variety of biological effects, such as anti-proliferation, anti-inflammation, and anti-oxidation, and has been regarded as one of the most promising agents from natural plants for future management of psoriasis (Müller et al., 1995). However, the poor solubility in aqueous and the low dermal permeability of BBR have hampered its clinical application. Freag and Torky’s group first reported monoolein (MO)-based BBR oleate-loaded (BBR-OL) LCNPs using the rapid and simple hydrotrope method (Freag et al., 2019). Compared to the other three cosolvents, ethanol, methanol, and tertiary butyl alcohol, polyethylene glycol 400 (PEG 400)/MO was shown to be the best polymer composition in terms of solubility, when a proper amount of Pluronic F127 (also called Poloxamer 407) was added as steric stabilizer. Moreover, the optimized BBR-OL-LCNPs as a liquid crystalline nano-reservoir enabled a sustained release and localized diffusion of BBR over time. Furthermore, the *in vivo* studies revealed that topical application of BBR-OL-LCNPs hydrogel significantly alleviated symptoms of psoriasis and reduced the levels of psoriatic inflammatory cytokines.

Compared with conventional liposomes, LCNPs have been proven to enhance the bioavailability and stability of loaded drugs. Nevertheless, when it comes to the pharmaceutical industry, the tremendous energy requirements and high cost of the liquid crystal preparation have been pointed out as obstacles for the generalized application of LCNPs (Lee et al., 2016).

#### 2.1.5 Transfersomes

Transfersomes, referred to as elastic or ultra-flexible liposomes, were firstly introduced by Cevc and Blume in 1992, and registered by the German company IDEA AG (Cevc and Blume, 1992). Transfersomes are structurally composed of phospholipids, edge activators (EAs), water, and no more than 10% ethanol as a vehicle (Fernández-García et al., 2020). The EA involved in transfersomes is usually biocompatible surfactants, such as sodium cholate, sodium deoxycholate (SD), polyols, Span 80, and Tween 80, functioning as membrane-softening agents to endow liposomes with elastic properties (Lei et al., 2013). With the addition of EA to completely replace cholesterol, transfersomes acquire an ultra-deformability that can spontaneously deform without damaging lipid structure when they are applied under non-occlusive conditions. Meanwhile, owing to the generated transdermal osmotic gradients and hydration force, transfersomes can pass through tiny pores even much smaller than the size of themselves, facilitating their transportation across the SC barrier to locate in deeper skin layers (Cevc and Blume, 1992; Jain et al., 2003). For these reasons, much more attention has been paid to the investigations of transfersomes for transdermal drug delivery and topical treatment of skin diseases.

It has been proven that different chemical structures of EA results in distinctive features and consequent behaviors of transfersomes, particularly the shape and size of vesicles, drug entrapment efficiency, degree of deformability, and the release and diffusion of entrapped drugs across the skin. For instance, Jain et al. compared the behaviors of transfersomes prepared with either SD, or Tween 80, or Span 80, designed at the hydrophilic/lipophilic balance of 16.7, 15, and 4.3, respectively, and loaded with lipophilic drug dexamethasone (Jain et al., 2003). In the following evaluations, the authors reported that among transfersomes derived from three types of EA, vesicle Tween 80 had a minimal degree of deformability, whereas transfersomes involving Span 80 demonstrated the greatest deformability with considerately higher drug entrapment efficiency. Hence, it can be concluded that a higher concentration of EA is associated with a lower drug entrapment and EA plays a crucial role in determining the skin permeability of elastic liposomes.

Specifically in the treatment for psoriasis, Sanna et al. designed and characterized BD-loaded transfersomes (BD-T) composed of phosphatidylcholine (PC) and SD or Tween 80 (Gizaway et al., 2017a). The optimized BD-T of particle size being 242.8 ± 1.2 nm was stable for at least 6 months at 4°C or 25°C. More importantly, when compared to the commercial BD cream, BD-T significantly improved the risk–benefit ratio showing a favorable therapeutic effect in patients with psoriasis. Likewise, to enhance the transdermal permeability of Tacrolimus (TAC), which is a lipophilic natural macrolide widely used in the treatment of psoriasis as a powerful immunosuppressant (Fereig et al., 2021), Parkash et al. constructed transfersomes using the rotary evaporation method with TAC, and tested the drug permeation followed by assessing its antipsoriatic efficacy (Ved Parkash et al., 2018). Compared to the conventional liposomes, a superior permeability was obtained when TAC was packaged into transfersomes, as assessed by pharmacokinetic and pharmacodynamic parameters, which was consistent with a gain in anti-psoriatic activities in both *in vivo* and *in vitro* studies.

In summary, transfersomes have been proven superior to conventional liposomes due to enhanced drug penetration and interactions with skin tissue. These encouraging results led to an increased number of clinical trials of transfersomes as drug carriers. It is noteworthy that, one main disadvantage of transfersomes as a drug delivery system is the difficulty in incorporating hydrophobic drugs without compromising their deformability and elastic properties. Besides, an increased amount of EA may result in reduced biocompatibility of transfersomes. Advances in techniques to increase the loading capacity of hydrophobic drugs into transfersomes require more endeavors.

#### 2.1.6 Ethosomes

Ethosomes, novel ultra-deformable nanovesicles essentially consisting of phosphides, with a high quality of alcohol in water, were described initially by Touitou et al. in 1996 (Touitou et al., 2000). Ethosomes could be considered as a special type of transfersomes, in which EA was replaced with ethanol. As constituents in ethosomes, phospholipids are used generally at a concentration of 0.5%–10%, while about 20%–45% of small-chain alcohols, such as ethanol, propylene glycol, and glycerol, would be specially introduced, functioning as enhancers for skin penetration. As its name implies, the addition of short-chain alcohols in ethosomes is essential since the presence of alcohols has greater effects on the size, zeta potential, entrapment efficiency, and stability of vesicles. In addition, by interacting with the polar head group of lipids, ethosomes can increase the fluidity of structural lipid bilayers of its own and the skin tissue, triggering drug release into deeper layers of the epidermis. Therefore, importing alcohol into liposomes serves to synergistically boost the phospholipid-enabled drug delivery across skin barrier in terms of quantity and depth.

Cyclosporine A (CyA), a potent immunosuppressive drug used in psoriasis, is considered clinically troublesome because of serious nephrotoxicity caused by systemic absorption. When used as a topical treatment, the high molecular weight of CyA (1,202 Daltons) dramatically limited its transportation across the skin barrier (must be under 500 Daltons). Recently, a comparative study on the transdermal delivery of CyA carried by liposomes, transfersomes, and ethosomes was reported by Carreras et al. (2020). With human heat-separated epidermis (HHSE) for evaluation, the results showed that although CyA-loaded ethosomes possessed the largest particle size of approximately 257 ± 1 nm, CyA ethosomes demonstrated the best skin permeability as compared to the others, i.e., ethosomes (∼60 μg/cm^2^) > transfersomes (below 10 μg/cm^2^) > liposomes (0 μg/cm^2^), experimentally supporting that the percutaneous permeability of ethosomes was superior to transfersomes and liposomes. Typically for antipsoriatic effects of ethosomes as topical therapies, Chandra et al. developed and assessed the potential of MTX-incorporated salicylic acid (SA)-loaded ethosomes gel using the cold method (Chandra et al., 2019). In the continuous monitoring for 24 h, MTX-SA-ethosomes gel exhibited a slow and prolonged release of MTX in contrast to MTX drug solution (26.13 ± 1.61% vs. 6.96 ± 0.06%), and more than 30% drug retention was detected in the skin exposed to MTX-SA-ethosomes. Consistent with drug release, MTX-SA-ethosomes formulation applied to the IMQ-induced psoriasis model significantly reduced the Psoriasis Area and Severity Index (PASI) score, showing a normal skin condition with a mild keratosis.

With the assistance of the solvent action from ethanol, ethosomes are inferred to be a better choice for drug delivery compared with transfersomes; however, risks of allergies may be concomitantly raised with the increase in the amount of ethanol. Apart from safety issues, the ethanolic core of ethosomes may probably evaporate under a relatively high temperature, for example, in the local inflammatory sites of psoriatic skin. Thus, further pharmacodynamic investigations and clinical efficacy would be highly required for clarification.

#### 2.1.7 Niosomes

Niosomes are vesicles presenting a similar structure to that of liposomes, while unlike liposomes, niosomes are formed by self-assembly of amphiphilic non-ionic surfactant with cholesterol, oriented into a bilayer structure with a neutral overall charge (Fang et al., 2008). As an alternative drug delivery system for conventional liposomes, niosomes are preferable mainly due to their smaller size, greater uniformity, and better reproducibility. Moreover, non-ionic surfactants, for example, Spans and Tweens, confer niosomes’ relative biocompatible, stable, and inexpensive nature, which is favorable for a large-scale production (Lakshmi et al., 2007). More relevant to psoriatic conditions, non-ionic surfactants could modify a horny layer to become a looser and more permeable organization, thereby increasing the residence time and local concentrations of drugs in the SC and epidermis (Sinico and Fadda, 2009). Furthermore, the amount of water presenting in the skin allows niosomes’ vesicles to pass SC in a diffusive way, which means that the penetration rate of niosomes largely depends on the concentration gradient created following the fusion or adhesion of niosomes to the skin surface.

Potential therapeutic and adverse effects of antipsoriatic drugs encapsulated in niosome have been explored. Lakshmi et al. entrapped 0.25% MTX in niosome vesicles and developed a chitosan gel (Lakshmi et al., 2007). In primary safety evaluations using the human repeat insult patch test (HRIPT), MTX niosomes did not show any significant irritation or sensitization in healthy human volunteers. Further antipsoriatic assessment of MTX niosomes was carried out through a double-blind placebo-controlled study on patients with localized psoriasis. After 12 weeks of treatment, clinical outcomes supported a stronger antipsoriatic efficacy of MTX niosomes compared to commercial MTX gel, and a significant reduction of PASI score from 6.24 ± 1.49 to 2.00 ± 0.14 was achieved. Also, a similar example has been reported for Acitretin (Act), an oral retinoid used only in the treatment of very severe resistant psoriasis because of its serious side effects on the risk of birth defects. Abu *et al.* developed Act niosomes made up of Span 60 and cholesterol using thin-film hydration (Abu Hashim et al., 2018). The stability of optimized Act niosomes was confirmed for at least 3°months at 4 ± 1°C, while Act niosomes were relatively unstable at room temperature (25 ± 1°C) showing drug leakage, which is possibly due to the oxidation and hydrolysis of the phospholipid bilayer. The specific therapeutic effects of Act niosomes as an antipsoriatic treatment was corroborated by both *in vitro* and *in vivo* tests. Act niosomes typically inhibited the highly proliferative HaCaT cells, a keratinocyte cell line derived from human, and the same consequence was reflected in a mouse tail model, showing significantly high orthokeratosis and decreased epidermal thickness without detectable side effect.

With the removal of phospholipids, niosomes overcome some disadvantages of lipid-based nanoparticles, such as low chemical stability against oxidation and temperature, strict preparation and stored conditions, and high cost. However, certain drawbacks associated with niosomes have been noticed, such as physical instability during preparation procedures due to aggregation, fusion, leakage, or hydrolysis of drugs.

### 2.2 Polymer-Based Nanoparticles (Polymeric NPs)

Polymeric NPs are colloidal systems made up of natural or synthetic polymers ranging from 10 to 1,000 nm, and can be loaded with both hydrophilic and hydrophobic compounds, which are entrapped inside or absorbed on the surface (Soppimath et al., 2001). Chitosan-based NPs from natural polymeric NPs and biodegradable aliphatic polyesters from synthetic polymers have been most frequently applied for topical skin delivery. Besides, a few US FDA-approved polymers have been widely employed, for example, alginate, albumin, hydroxyapatite, and hyaluronic acid from natural polymers, as well as poly(glycolic acid) (PGA), polylactide (PLA), poly(lactic-co-glycolic acid) (PLGA), and PEG from synthetic polymers. In the preparation of nanoencapsulation with these polymers, the most commonly applied methodology includes nanoprecipitation, solvent evaporation, emulsification/solvent diffusion, emulsification/reverse salting out, and *in situ* polymerization technique (Crucho and Barros, 2017). Polymeric NPs are attractive for topical drug delivery because they offer promising characteristics such as tunable size, protection for labile molecules, modified surface with ligands, and controllable drug release. For instance, the drug-loaded polymeric NPs with surface modifications by peptides, aptamers, or antibodies could potentially target specific cells or tissues for targeted drug delivery. Likewise, the drug release can be controlled by modulating the drug-to-polymer ratio, or composition and molecular weight of polymers.

Epigallocatechin gallate (EGCG), the most abundant catechin in green tea extract, has been demonstrated to have antioxidant and anti-inflammatory effects against a variety of diseases. However, due to its extremely low membrane permeability and requirement for transporter-mediated intestinal efflux, EGCG has unsatisfactory oral bioavailability. To tackle this problem, Chamcheu et al. fabricated soluble EGCG nanoparticles based on positively charged chitosan (nanoEGCG) using the gelation method, which provides a superior bio-adhesive property (Chamcheu et al., 2018). The nanoEGCG showed a 50% quick release of EGCG after 6 h, and then a further release of 100% EGCG was detected after 24 h. Compared to free EGCG, the inhibitory effects of nanoEGCG on human keratinocyte hyperproliferation induced by IL-22 were shown to be more than 4-fold efficient. Consistent with *in vitro* results, delivery of nanoEGCG in an IMQ-induced psoriasis mouse model was found to be 3 times higher than free EGCG.

Similarly, using a conventional anti-solvent method, Sun et al. developed Cur-PLGA nanoparticles (Cur-PLGA-NPs) in various sizes: small, 48.89 ± 0.19 nm; relatively large, 152 ± 1.39 nm (Sun et al., 2017). Through *in vitro* skin permeability assay using excised (human/porcine) psoriatic skin, the small-sized Cur-PLGA-NPs showed the highest skin Cur accumulation, being 1.31 ± 0.07 μg/cm^2^. In agreement with the efficiency in skin permeability, the symptoms and biomarkers associated with psoriasis were significantly reduced by the small-sized Cur-PLGA-NPs, shown as improved PASI score and reduced serum levels of cytokines.

With the progress in the development of polymeric NPs, a diverse type of drug-loaded polymeric NPs could be implemented based on the properties of polymer and drugs, as well as the preparation methods applied. Apart from advantages in formulations, a variety of reports have suggested a potential application of polymeric NPs as topical carriers for antipsoriatic treatment, supporting the idea that polymeric NPs are becoming an important strategy to control drug delivery across skin. However, when compared to other developed nano-formulations, polymeric NPs were shown to be more accumulated in SC of inflamed skin, thus having limited capacity in the enhancement of skin permeation. Hence, advanced techniques that enable polymer-based NPs to be a more effective and targeting transdermal strategy would lead to an increasing interest in the evolution of drug delivery systems.

### 2.3 Metal-Based Nanoparticles

MNPs are solid colloidal metal particles for biomedical applications, with size ranging from 1 to 100 nm. MNPs can be synthesized by various methods, for example, the most used chemical reduction method and the most popular green chemistry approach, which utilizes cost-effective and environmentally safe natural extracts.

Due to their unique characteristics, namely, magnetism, large surface area-to-volume ratio, and ease in surface modification, as well as a diversity of biological effects such as antimicrobial, anti-inflammation, and anticancer, a variety of MNPs have been investigated for drug delivery. Typically, MNPs are perfectly suitable for topical drug delivery, because MNPs not only exert biological effects by themselves but also create a synergistic effect with other anti-inflammatory agents. So far, several MNPs like gold, silver, selenium, and platinum nanoparticles have been explored in treating psoriasis.

Bessar et al. designed water-soluble functionalized gold nanoparticles (AuNPs) containing MTX for topical delivery (Bessar et al., 2016). Small-sized (4 ± 1 nm) AuNP-MTX obtained in this study demonstrated a satisfactory stability for 6 months at 4°C, and a stronger inhibitory effect on keratinocytes compared to free MTX. A fast release of MTX of about 80% in an hour and 95% in 24 h was detected in this system, which indicates that the amount and duration of the excipients applied on the skin can be potentially reduced. Moreover, by using ultraviolet-to-visible (UV-Vis) spectroscopy, the authors reported that MTX in the functional AuNPs was absorbed not only in epidermis but also in dermis of C57BL/6 mouse skin, which was greater than the skin penetration of mice exposed to MTX alone. Further investigations carried by Fratoddi et al. verified the antipsoriatic efficacies of topically applied AuNPs-MTX using an IMQ-induced psoriasis-like mice model, showing the therapeutic properties of AuNPs-MTX in both clinical responses and biological indicators, such as reduced epidermal thickness, cell proliferation, and inflammation (Fratoddi et al., 2019). Besides AuNPs, the antipsoriatic effects of NPs derived from other metals have also been evaluated. By using green technology, Crisan et al. prepared silver nanoparticles (Agnese) carrying polyphenol-rich extracts (*Cornus mas*) and compared the therapeutic efficacy with its counterpart prepared from AuNPs (Crisan et al., 2018). In both *in vitro* assessment of pro-inflammatory macrophages and *in vivo* evaluations of human psoriasis plaques, the two complexes consistently diminished the associated symptoms and suppressed the levels of inflammatory cytokines without treatment-related side effects, while the smaller sized Au-NPs-CM had a better permeability capacity and consequently are more effective than Ag-NPs-CM.

Although a superior anti-inflammatory efficacy has been addressed in MNPs, several studies have reported that metallic NPs may trigger skin sensitization, manifested as allergic and irritant dermatitis. The metallic ions released from metallic NPs were thought to be the major reason that causes skin immunoreaction (Niska et al., 2018; Wang et al., 2018). It is appealing that evidence has specified that metallic NPs synthesized by green synthesis exert less cytotoxic effects than those synthesized chemically. Debates regarding the cytotoxicity caused by interactions of metallic NPs and skin cells are ongoing, while green synthetic approaches may likely propel MNPs forward.

### 2.4 Nanofibers

Nanofibers are highly competent for drug delivery due to their high surface area-to-volume ratio, increased porosity, favorable mechanistic properties, and flexible functionality compared to conventional micro-fibrous materials. Similar to native extracellular matrix, nanofibers interconnect to form a scaffold, which is endowed with excellent architecture for liquid absorbance, moisture balance, and gas permeability (Rasouli et al., 2019). More intriguingly, nanofibers are often used cooperatively with encapsulated nanoparticles for the benefit of both, promoting synergistically the recovery of psoriatic lesions. Currently, nanofibers in medicine are frequently synthesized from polymeric materials, being either natural or synthetic, or a combination of both. In contrast to natural polymers, which have better biocompatibility and lower immunogenicity, synthetic polymers provide greater flexibility and modification potentials (Goyal et al., 2016). In addition to polymeric materials, synthetic technologies employed to produce nanofibers have an essential place that impact the properties of nanofibers. So far, a variety of methods have been developed, ranging from conventional technologies, such as electrospinning (ES), self-assembly, polymerization, and template-based synthesis, to emerging strategies like solution blow spinning, centrifugal jet spinning, and electrohydrodynamic writing. Each of these materials and techniques has specific importance and application, based on individual clinical scenarios.

Electrospun fibers and nanocellulose, typically bacterial nanocellulose (BNC), are the two preferred nanofibers for the treatment of psoriasis by adopting electrostatic spinning technology and microbial fermentation technology, respectively. Synthetic polymers such as polyethylene oxides (PEO) for ES and microbe-derived highly purified natural polymers for BNC are frequently used as building blocks (de Lima Fontes et al., 2018; Afsharian and Rahimnejad, 2021). Although these nanofibers in the form of topical patches are beneficial in terms of quantitative drug delivery, convenient application, and patient compliance, both have encountered several flaws that require improvement.

#### 2.4.1 Electrospinning

Of all the current strategies available for the fabrication of nanofibers, electrospinning is the most frequently adopted method since it is relatively simple, user-friendly, and cost-effective. During the ES process, a viscous polymer solution is squeezed from a syringe into a drop while strong electrostatic forces are applied to overcome the cohesions of polymer and to induce charge within the polymer and needle. When the charge repulsion force exceeds the surface tension of polymer, a jet will be initiated from the needle tip, referred to as a “Taylor cone,” that creates droplets in an extremely thin, elongated form with very high surface area. As the solvent rapidly evaporates from these droplets, nanosized fibers are formed and attracted to an oppositely charged or grounded collector. The benefits of ES are realized in tissue engineering, biosensors, filtration, wound dressings, drug delivery, and enzyme immobilization. In the case of drug delivery, different drugs or compounds can be added to the polymer solution during synthesis, and thus become incorporated into the nanofibers during this process. Moreover, in contrast to conventional methods in the industry like hot melt extrusion or spray drying, the ES that applies electricity for nanofiber dryness has less energy loss and is more environmentally suitable for maximum retention of drug properties. Furthermore, precise control of the areas being exposed to drugs and the rate of drug release over a desired period can be achieved through degradation/adsorption of nanofibers, within which drugs are packaged. In addition, ES can solubilize drugs in liquid phase, and then covered by a solid ultrafine fibrous scaffold. This format is preferred in driving higher loading and uniformed distribution of poorly soluble drugs into nanofibers, especially under the rapid evaporation process of ES.

The efficient delivery of drugs by ES mainly relies on passive diffusion through skin appendageal routes by establishing a high concentration gradient of drugs across the skin. Despite providing a scaffold, ES is favored because of its assistance in biocompatibility, drug stability, and a possibility for multi-drug encapsulation. Brooker et al. designed anti-inflammatory fibrous sheets that were synthesized from a propylene sulfide monomer *via* nanoemulsion polymerization before being electrospun into PEO fibers. This nanofiber exhibited high cytocompatibility when applied to human dermal fibroblasts (Brooker et al., 2021). On the occasion of psoriasis, the commercially available poly(methyl vinyl ether-alt-maleic ethyl monoester) (PMVEMA-ES) nanofibers have been designed as a delivery vehicle for three compounds, namely, salicylic acid, methyl salicylate, and capsaicin, which constitute the main agents to alleviate psoriatic symptoms. Being loaded into PMVEMA-ES with an encapsulation efficiency of approximately 100%, both salicylic acid and capsaicin were stably maintained while methyl salicylate remained approximately 60% after 15 days of encapsulation. This study provides evidence that ES is suitable for the creation of skin adhesive dressings for psoriasis, allowing encapsulation of multiple compounds (Martínez-Ortega et al., 2019).

Currently, ES nanofibers for psoriasis are mainly based on a simple blending method, in which the release of drug is mediated through simple surface diffusion and pores caused by the degradation of fibers. The drawbacks of simple blending ES, such as rapid denature of sensitive drugs and burst release of loaded components, requires more sophisticated modifications. To further satisfy ES applications in psoriasis, the variety of possibilities in the selection of polymers, drugs, and different types of ES offer potential prospect. For example, the core-shell structure of nanofibers formed by using coaxial ES holds a double-layer shielding effect, in which unstable components may be protected in the core and the sudden release of drugs would be slowed down until the degradation of the shell layer. Particularly in psoriasis, which involves complex pathological alterations of skin, multi-drug loading and sequential drug release corresponding to the lesions of psoriasis can be realized, which could be designed as keratolytics being embedded in the shell and anti-inflammatories being embedded in the core. In addition, certain volatile agents may experience a rapid evaporation and thus loss before application. This phenomenon can be solved by cooperating ES with other nanomaterials, such as hydrogel. On the one hand, the hydrogel can assist the delivery system to control drug release and provide a moisturizing effect for psoriatic skin, and on the other hand, ES can improve the ductility of the hydrogel.

#### 2.4.2 Nanocellulose

Cellulose is chemically a linear homo-polysaccharide polymer, consisting of glycan chains that are linked together by β-1,4-glycosidic bonds. Nanocellulose refers to cellulose particles with at least one dimension less than 100 nm, which are typically used in the form of cellulose nanofibers (CNF), cellulose nanocrystals (CNC), and microbial nanocellulose (MNC). Though all the three types of cellulose are nano-structured, they are distinct in terms of shape, size, and composition. Both CNF and CNC are plant-based, while MNC could be derived from algae, fungi, and bacteria. For the fabrication of CNF and CNC, disintegration of plant cellulose by mechanical, physical, and chemical approaches or a combination is often involved. Compared to the top-down methods involving the breakdown of the bulk materials into nanosized structures or particles, MNC is bottom-up synthesized nanofibers, using methods such as fermentation (de Amorim et al., 2020). Compared to plant cellulose, MNC has a high aspect ratio, which renders MNC a greater surface area per unit mass. This feature, together with its great hydrophilic nature, enables MNC as a competent liquid loading material. Meanwhile, the biocompatibility of MNC makes it an attractive candidate for a wide range of biomedical applications (Iguchi et al., 2000).

Among MNCs, BNC is synthesized by certain microbial genera, belonging to the Gram-negative non-pathogenic bacterial genera like *Rhizobium*, *Xanthococcus*, *Pseudomonas*, *Azotobacter*, *Aerobacter*, and *Alcaligenes*, and the strains from the *Komagataeibacter* genus are the most recognized producers. The BNC is commonly produced by static, shaking, or agitation fermentation with a two-step process, namely, polymerization and crystallization. Initially, glucose residues in the bacterial cytoplasm polymerize to linear glucan chains, which are extracellularly secreted. Meanwhile, the developed chains are crystallized to microfibrils, followed by consolidation of microfibrils to a highly pure tridimensional porous network (Koizumi et al., 2008). In recent years, BNC has earned enormous interest because of its green processing, water holding capacity, thermal properties, and mechanical durability, which make BNC a superior biomaterial for skin diseases. Specifically, the moist environment of BNC renders an effective physical barrier against pathogens, and its ability to absorb exudates along with their air permeability and comfortability constitute other essential benefits that are suitable for pustular psoriasis or lesions with exudative tendencies. Likewise, the resemblance of BNC with cellular matrix, such as collagen, and its appreciable water retention capability further make it clinically relevant for the treatment of dry skin condition as happens in psoriasis.

When administered as a drug delivery carrier for the treatment of psoriasis, the favorable properties of MNC can be additionally enhanced by appropriate chemical modification. Almeida et al. reported that BNC membrane incorporated with glycerin had a statistically higher moisturizing effect than BNC without glycerin, an effect highly required for psoriasis and atopic dermatitis (Almeida et al., 2014). In addition to the moisturizing effect, improvement of skin penetration property addresses the potentials of natural wood cellulose as drug carriers. In a Cur-loaded lipid-hybridized cellulose nanofiber system, lipid-hybridized cellulose potentiated a more than twofold increase of Cur deposition to the epidermis of an IMQ-induced psoriatic mouse, compared with the films without lipids (Kang et al., 2018). Similarly, Fontes et al. successfully developed a BNC/carboxymethylcellulose (BNC/CMC) biocomposite that carried MTX, as an alternative for the topical treatment of psoriasis (de Lima Fontes et al., 2018). Despite raising the amount of drug loaded, BNC preferentially permits a long-term drug release, since the tridimensional network of BNC membranes generates a relatively lower diffusion of the drug molecules as compared to commercial gel (Silva et al., 2014).

The bottleneck for the development of BNC turns out to be the lack of efficient fermentation systems, high cost on an industrial scale, and difficulties in controlling structure and properties through standard manufacturing and digital techniques. Alternative low-cost carbon sources, such as agricultural wastes (Jozala et al., 2015), have been considered to substitute the growth media for BNC generation, which accounts for up to 30% of the production cost (Rivas et al., 2004). However, several issues seem to be associated with the implementation of alternative carbon sources, as crystallinity, O_2_ and H_2_O transmission, and the degree of polymerization of BNC have been shown to be simultaneously affected though productivity was augmented (Jozala et al., 2015; Salari et al., 2019). The optimization of BNC properties, increase of yields, reduction of costs, and selection of appropriate industrial fabrication lines are the main future goals to promote BNC application.

### 2.5 Other Nanocarriers

#### 2.5.1 Micelles

Micelles are formed by self-assembly of amphiphilic molecules including surfactants, polymers, and copolymers in an aqueous system through the hydrophobic effect. Micelles often present as a spherical structure less than 100 nm in diameter because the least amount of energy is needed (Lu et al., 2018; Larsson et al., 2021). The structure of micelles contains a hydrophobic core and a hydrophilic shell to carry lipophilic drugs in the core and hydrophilic drugs in the shell, which is suitable for drug administration. Being used as a drug carrier in dermatology, micelles are mainly involved in improving the stability, water solubility, and skin penetration of poorly soluble drugs. According to the available literature, the change of surface electronic property may influence the skin penetration by either interacting with helical filaments of the SC or interacting with the keratin fibrils of the cornified cells, or inducing fluidization of the SC lipids (Walters et al., 1988; Kitagawa et al., 2000; Dragicevic-Curic et al., 2010; Morris et al., 2019; Okasaka et al., 2019; Strati et al., 2021). Further skin diffusion studies have demonstrated that in terms of skin permeation, the amphoteric emulsifiers were superior to non-ionic surfactants, but inferior to anionic emulsifiers (Karande et al., 2005; Pham et al., 2016). In addition, different patterns of drug release from micelles have been proposed to be dependent on the loading method and drug location; e.g., drugs loaded by physical entrapment or located in the shell/interface are released by diffusion, while drugs loaded by chemical conjugation or located in the center are released by micelle degradation or surface erosion (Kosakowska et al., 2018; Supasena et al., 2020).

Mycophenolic acid (MPA), Ciclosporin A (CsA), and TAC are conventional immunosuppressant medications used for psoriasis; however, the low aqueous solubility has greatly restrained their therapeutic applications. By conjugating MPA with poloxamer 407 (P407, also referred to as Pluronic F-127), an amphiphilic synthetic triblock copolymer consisting of a hydrophobic poly(oxypropylene) (POP) between two hydrophilic poly(oxyethylene) (POE) blocks (Jones et al., 2009), Supasena et al. investigated the antipsoriatic activities of P407-MPA micelles. The P407-MPA micelles structurally exposed its POE chain to the aqueous environment and therefore protected conjugated MPA inside the core. As a result, P407-MPA micelles achieved an improved aqueous solubility and biological activity, with a sustained MPA release in human plasma and more potent antiproliferation activity in an *in vitro* psoriasis model (Supasena et al., 2020). In order to avoid severe systematic side effects induced by oral CsA administration, Lapteva et al. attempted to encapsulate it into methoxy-poly(ethylene glycol)-dihexyl substituted polylactide (MPEG-dihex-PLA) micelles and developed single-component aqueous formulations for topical delivery to psoriatic skin. More intriguingly, these nanoscaled and spherical micelles increased the aqueous solubility of CsA by up to 518-fold (Lapteva et al., 2014b). In a similar way, to enhance the cutaneous bioavailability of TAC, this hydrophobic macrolide was loaded with MPEG-dihex-PLA diblock copolymer to form micelles. Compared to TAC ointment, TAC-based micelles resulted in significantly greater TAC deposition in human skin, and the increase in cutaneous drug levels was proved to be due to the improved transdermal delivery of TAC (Lapteva et al., 2014a). Likewise, Jin et al. conjugated the photosensitizer zinc phthalocyanine (ZnPc) with the PEG chain of Brij 58 (ZPB), an inert surfactant that does not affect protein bioactivity and conformation (Yue et al., 2020). This nanostructure kept a shell-core organization with the ZnPc located at the core and the PEG chains located at the shell. Using a guinea pig psoriasis model, the combination of light and ZPB showed a nearly cured antipsoriatic effect, as confirmed by histopathology (Jin et al., 2015).

The micelle is susceptible to the influence of the *in vivo* environment like pH, temperature, or reduction–oxidation reactions, which may result in structural deformation and subsequent release of loaded drugs or induction of drug resistance. In accordance with this property, many pH-triggered and thermosensitive micelles have been designed to achieve targeted drug delivery (Bae et al., 2005; Guo et al., 2018; Wang N. et al., 2020). Therefore, a micelle is more suitable for topical administration or in combination with other polymer materials when controlled drug release is desired (Wen et al., 2017; Chen et al., 2019).

#### 2.5.2 Nanoemulsions

NEs are known as nanocolloidal carriers with droplet size ranging from 20 to 500 nm. NEs are formed in the dispersion of two immiscible liquids (frequently water and oil) that are not soluble in one another and in which no phase boundary is visible (Solè et al., 2006; Singh et al., 2017). Three types of NEs have been developed according to specific applications, namely, oil-in-water (o/w) emulsions, water-in-oil (w/o) emulsions, and bicontinuous NEs. Methods used for NE preparation are high-energy emulsification, low-energy emulsification, or a combination of both. A small particle size of NEs can be easily achieved with high-energy methods, whereas thermolabile molecules cannot be used. Unlike high-energy emulsification, low-energy emulsification is based on the physicochemical properties of components, which is prevailing when expensive or sophisticated manufacturing equipment as required for high-energy methods are unavailable (Bernardi et al., 2011). The produced o/w or w/o NEs can be formulated into a variety of topical formulations, such as liquids, sprays, and creams, transporting highly hydrophilic or hydrophobic components (Wang et al., 2007; Colucci et al., 2020).

The compositions of the oil phase in NEs are generally selected based on both the biocompatibility and inherent activity of oil, and the solubility of entrapped drugs. Capryol 90 and oleic acid are the common candidates preferentially applied in the preparation of micelles for psoriasis treatment. Selection of surfactants and cosurfactants requires considerations of both drug solubility and the emulsification capacity of surfactants, and Tween 80 and squalene constitute the generally applied surfactants (Musa et al., 2017; Ramez et al., 2018; Dadwal et al., 2020a; Khatoon et al., 2021). Both oil phase and surfactants can disrupt skin lipid bilayers and SC and thereby facilitate skin permeation of entrapped compounds.

The efficiency of transdermal drug absorption of w/o NEs is promoted through skin appendages *via* increasing the solubility of drugs and generating a greater concentration gradient between NEs and the skin (Wu et al., 2001). Scalp psoriasis is an intractable medical problem, conventional therapies for which rely on long-term topical corticosteroid administration (Turpeinen, 1989; Renton, 2014). To avoid the side effects of topical corticosteroids, Langasco et al. formulated a Clobitasol Propionate (CP)-loaded w/o NEs system. Construction of nano-emulsified CP contributed to enhanced absorption and penetration of corticosteroids specifically in SC and the epidermis. By intensifying CP accumulation and retention in the upper skin layer, a desired merit for psoriatic scalp and reduced systemic side effects were obtained (Langasco et al., 2018). As for o/w NEs, its low surface tension contributes to skin hydration and changes in SC structure, and consequently makes the penetration of drugs through the skin barrier easier (Kim et al., 2014). For example, Musa et al. developed Cyc-loaded o/w NEs whose oil phase was composed of a mixture of nutmeg and virgin coconut oil. Due to a higher content of fatty acids in the mixed oil solution, the emulsified Cyc showed not only an increment in drug loading but also an improved skin hydration in healthy volunteers (Musa et al., 2017). In a similar way, to promote permeation of lipophilic Cur into SC, Yousef et al. developed a series of w/o NE systems by combining different proportions of oil, surfactant, xanthan gum, and water content. The optimized formulations for Cur enhanced transdermal Cur delivery through NE-based increase in both the SC solubility and SC diffusivity of Cur (Yousef et al., 2019). Compared to w/o NEs, o/w NEs have been proven to behave better in the transdermal delivery field due to structural stabilities and skin permeation capabilities (Colucci et al., 2020). Additionally, some specially designed NE systems are preferred as moisturizers for daily skin care of psoriatic patients. For instance, rice bran oil has unsaponifiable fractions that contain high levels of antioxidant-rich components and has been widely formulated in cosmetics. Using rice bran oil, Bernardi et al. developed o/w NEs *via* a low-energy emulsification method. When applied to the skin of volunteers suffering from psoriasis and atopic dermatitis, the NEs increased skin hydration and maintained normal skin pH (Bernardi et al., 2011). However, the low viscosity of NEs might not be advantageous for topical applications. This could be practically overcome by a hydrogel-thickened procedure. Algahtani et al. encapsulated retinyl palmitate (RP)-loaded NEs into hydrogel systems as a secondary vehicle, which assisted the permeation and release of RP from NEs, and consequently minimized the side effects of RP, such as skin irritation (Algahtani et al., 2020).

The formulation of NEs generally demands the participation of high concentrations of surfactants or cosurfactants, most of which have systemic toxic effects when used over a large skin area (Emanuele and Balasubramaniam, 2014). Besides, when the NEs are diluted, their structure is often destroyed because the original ratio of each phase is altered and thus no longer fits the formation of NEs (Rai et al., 2018). Decreasing the use of surfactants and cosurfactants, or finding efficient and less toxic alternatives, as well as optimizing the process to maintain the morphological integrity and stability of NEs droplets are critical for the next advanced steps.

#### 2.5.3 Nanogels

Hydrogels are crosslinked polymeric and tunable porous structures that have a high affinity for water but do not dissolve in water. The feature of being highly absorbent or capable of holding a large amount of water while retaining an intact structure has made hydrogels ideal candidates in extensive biomedical applications. The nanoscale synthesis of hydrogels results in nanogels, which restricts the swelling potential but dramatically enhances the mechanical stability and drug delivery functionality of hydrogels. Compared to hydrogels, nanogels tend to be more frequently used as a drug delivery system due to their relatively high drug encapsulation capacity, uniformity, tunable size, ease of preparation, and minimal toxicity.

Based on the crosslinked structure, nanogel networks derived from either natural or synthetic polymers can be mainly divided into two categories: chemically and physically crosslinked nanogels. Chemical crosslinks by covalent bonds lead to a permanent, stable, and rigid polymer network, whereas physical interactions that self-assemble through weaker linkages by non-covalent bonds are shaped by polymer chain entanglements or by physical connections, such as electrostatic, hydrogen bonding, van der Waals, and hydrophobic interactions (Neamtu et al., 2017). Likewise, the methodologies for nanogel synthesis can be classified into chemical and physical routes, with the former being the most developed technique for nanogel production. The versatility of chemical strategies has been established, including emulsion polymerization, controlled/living radical polymerization, click chemistry, and photo-induced crosslinking. Candidate materials are usually low-molecular-weight monomers, polymer precursors, or polymers with specific terminal or pendular reactive groups. Compared to chemical methods, physical crosslinking requires mild reactions, mainly in water with reduced adverse effects, and the size of nanogels can be more flexibly regulated by modulating polymer concentration and parameters, such as temperature and pH. The synthetic materials for this approach are molecules presenting a hydrophilic framework and several grafted hydrophobic moieties, or protonatable groups, like polysaccharides, cholesterol, and gelatin. More innovative technologies, including microfluidics and 3D printing, have been developed for the purpose of high efficiency, high controllability, low cost, and scalability (Mauri et al., 2021)*.*

Conventional nanogels have an excellent hydrophilic nature that, however, limits the efficiency of hydrophobic drug encapsulation. Therefore, suitable engineering of polymers was adopted to allow incorporation of poorly soluble drugs, designed as amphipathic nanogels (Bewersdorff et al., 2019). By this method, nanogels open a new avenue for transporting insoluble drugs, which, on the one hand, enhances the solubility and stability of loaded components and, on the other hand, potentiates the cellular uptake or penetration of lipophilic drugs. Following this concept, nanogel, as a matrix for topical drug delivery, has been accordingly modified by incorporating with auxiliary materials. For example, certain clinically used medications and herbal extracts effective in releasing psoriasis, like methoxsalen, MTX, Cur, and BBR, are hydrophobic molecules being unable to be directly loaded into a hydrophilic carrier (Ali et al., 2008; Sun et al., 2017; Freag et al., 2019). To tackle the barrier of percutaneous penetration of drugs in the treatment of psoriasis, Barradas et al. produced hydrogel-thickened NEs for lipophilic 8-methoxypsoralen using polysaccharide chitosan, which was incorporated to overcome the low viscosity of conventional NEs (Barradas et al., 2018). Freag et al. designed a hydrogel comprising a liquid crystalline nanoparticulate (LCNP) reservoir of hydrophobic BBR blended with oleate (Brb-OL). This solubility modification for BBR ensured a threefold increase of drug accumulation and a tenfold augmentation of drug permeation compared to crude BBR and hence alleviated inflammatory cytokines presented in psoriasis (Freag et al., 2019). To improve the conditions of poor aqueous solubility and chemical stability, Cur-loaded PLGA nanoparticles (Cur-NPs) were fabricated and tested by topical administration in an IMQ-induced psoriasis-like mouse model. The resultant Cur-NPs hydrogel was superior to lipophilic cur as antipsoriatic interventions, associated with improved solubility, sustained drug release, and enhanced drug penetration across the skin (Sun et al., 2017). In an animal skin permeation study, the remodel of plain MTX hydrogel to MTX-liposome hydrogel led to enhanced delivery of MTX into skin layers, and a slow and sustained release of MTX from liposomes reduced the accumulation of MTX in epidermal layers over long periods of time (Ali et al., 2008). This set of results reveals that while preserving the efficacy of drugs, the control of drug release and reduction in systemic side effects could be simultaneously achieved by using colloidal drug carriers, such as liposomes and nanoparticles. Supportingly, in order to alleviate skin atrophy and vasodilation induced by steroid hormone in the treatment of psoriasis, Kumar et al. successfully fabricated a corticosteroid CP-loaded cyclodextrin nanosponge, which was further embedded into hydrogel. This formulation was shown to augment the 45-fold water solubility of CP, in addition to enhanced anti-psoriasis action, controlled drug release, and neglectable cytotoxicity (Kumar et al., 2021). In the case of macromolecules, it remains impossible to cross an unimpaired SC through passive diffusion in a colloidal carrier system. By employing laser ablation, the topical cutaneous delivery and controlled release of macromolecules were enhanced. ETA is a biological medical product of 150 kDa used to treat autoimmune disease including psoriasis. Due to its large molecular weight, ETA is far from being an ideal candidate for topical delivery. However, when it was dissolved in polyvinylpyrrolidone gel, with the assistance of Er:YAG fractional laser ablation (FLA), a deeper diffusion of ETA into the epidermis and dermis was obtained, and a controlled drug delivery *via* modulating the laser parameters could be achieved (Del Río-Sancho et al., 2020).

While nanogels are considered advantageous over other drug delivery systems, as discussed above, nanogel used for drug delivery generally has poor loading capacities for hydrophobic drugs, and nanogel cannot enhance percutaneous penetration as it is, along with the fact that nanogel is not preferred for mechanical and self-healing properties; thus, a combination of other polymeric materials such as liposomes, micelles, and ES would be employed. Additionally, although the manufacturing process of nanogel is not very pricey, it is rather expensive to remove the surfactant and the solvent at the end of the preparation process. Adverse effects may occur if any traces of polymers or surfactant remain in the body (Urakami et al., 2013).

## 3 Physical Transdermal Drug Delivery Systems

Amidst the progress of nanotechnologies, physical approaches facilitating drug penetration through skin barriers have been continuously developed, including non-invasive and invasive delivery.

### 3.1 Non-invasive Delivery

#### 3.1.1 Iontophoresis

Simply referred to as “movement of ionic drug molecules with electric current”, IP is a non-invasive drug delivery system that uses a low-intensity electric current (0.3–0.5 mA/cm^2^) (Fukuta et al., 2020). IP allows controlling the delivery rate in a preprogrammed manner fulfilled by two electrodes. The electrode containing the drug system is termed active electrode, whereas the return electrode (located adjacent to the active electrode) completes the circuit. Factors affecting the efficiency of IP include current density, pH, drug concentration, molecular size of drug, and method of current application (continuous or pulse current) (Wang et al., 2021). Accumulating experimental evidence shows that iontophoretic transport of drugs across the skin occurs through appendageal and intercellular pathways, mainly mediated by two mechanisms: (1) a flux of ions generated by electric potential across the skin; (2) the electroosmotic or convective flow that occurs in the “anode-to-cathode” direction due to the net-electrical charge of the skin (Burnette and Ongpipattanakul, 1987; Guy et al., 2000; Guy et al., 2001). In particular, it was reported that Ca^2+^ influx into skin cells and subsequent intracellular signal activations are induced *via* IP, which leads to a decrease in expression of gap junction protein connexin 43 and depolymerization of tight junction-associated polymerized actin, resulting in weakening of intercellular connections (Hama et al., 2014).

One of the advantages of IP-mediated drug delivery is that intradermally delivered drugs *via* IP are gradually released into the systemic circulation and maintained at a certain level upon sustained transportation of drugs from the skin to circulation (Takeuchi et al., 2017). In practical utilizations, biological macromolecular drugs like antibodies, proteins, peptides, and oligonucleotides, which are normally administered by invasive intravenous or subcutaneous injection with needles, have been confirmed to be suitable for IP method in transdermal delivery (Kanikkannan, 2002). Fukuta et al. have reported that non-invasive IP could implement the transdermal delivery of antibody or anti-TNF-α fusion protein ETA in an IMQ-induced psoriasis rat model. Through the assistance of fluorescein isothiocyanate (FITC) labeling, the authors provided evidence that antibodies, which are very large molecules with high hydrophilicity, could be successfully delivered up to 80% into skin tissue by IP. More intriguingly, IP administration of ETA ameliorated psoriatic epidermis hyperplasia more significantly than needle injection and concurrently avoided the invasive injuries. To support the above findings, intradermal delivery of hydrophilic macromolecular drugs, including interfering RNA (siRNA; M. W. 12,000) and CpG oligo DNA (M. W. 6,600), *via* IP has been established, which was proved to exert their individual biofunctions *in vivo* (Fukuta et al., 2020).

Though nanomaterials have greatly improved the efficiency of topical drug delivery, the treatment for nail psoriasis and arthritic psoriasis remains an inadequately addressed issue. It has been demonstrated that IP could improve drug permeation over passive formulations by up to 37 times in onychomycosis or nail fungus (Nair et al., 2011). A retrospective study on nail psoriasis treated with dexamethasone IP weekly for at least 3 months demonstrated that 81% of patients showed an improvement in psoriatic nails (Van Le and Howard, 2013). Similarly, the powerful transdermal effect of IP has endowed itself with a promising application for arthritic psoriasis (Patel and Wairkar, 2019). In addition, with great progress and application of biologic therapies for psoriasis, the administration of biological macromolecular drugs by IP helps to avoid problems introduced by traditional needle injection, such as inflammation and injuries (Fukuta et al., 2020).

Because IP does not basically alter the skin structure itself, it is mostly suitable to small molecules that are charged and some macromolecules up to a few thousand Daltons; therefore, the transdermal effect of large molecules is to some extent limited. Moreover, since the dosage of drug scales with the amount of charge delivered to the skin, the ability of IP depends on the current applications, which are restricted by the battery capacities of IP (Prausnitz and Langer, 2008).

#### 3.1.2 Ultrasound (Sonophoresis)

Sonophoresis is a technique that employs ultrasound energy as a mechanical enhancement tool to drive systemic delivery of therapeutic agents. Ultrasound is defined as sound with a wave frequency above 16 kHz, which is greater than the upper limit of human hearing ability. Based on the frequency used for ultrasound, the ultrasonic waves can be classified into three categories: low frequency (<100 kHz), intermediate frequency (100–700 kHz), and high frequency (>1 MHz) (Rich et al., 2014; Daftardar et al., 2019). The mechanistic aspects of biological consequences of ultrasound are associated with thermal effects and cavitation mechanisms. Thermal effects occur as the ultrasonic beam travels through the medium, which absorbs the sonic energy and converts into heat (O'Brien, 2007). The augmentation of local temperature is the predominant effect of low-frequency ultrasound and varies in proportion to the intensity and duration of ultrasound. Cavitation is the effect due to gas bubbles produced in the ultrasonic field. Cavitation mechanism occurs when the ultrasound wave propagates through the liquid, where regions of compression and rarefaction are created, resulting in alternations in pressure. Pressure changes cause a dynamic formation and oscillation of gas bubbles, being either stable or inertial (Rich et al., 2014). Typically, in inertial cavitation, the rapid expansion and violent collapse of microbubbles in a liquid medium at higher acoustic pressures result in the release of shock waves, leading to structural alterations in the surrounding tissues.

Naturally, the SC of skin is composed of corneocytes interspersed in a laminate of keratin and inter-corneocyte lipid. Corneocytes are differentiated dead keratinocytes, which exist from the basal layer to the granular layer where they transform into the corneocytes of the SC. As the most important layer of skin that provides substantial protection from foreign molecules, SC equivalently constitutes the gatekeeper for transdermal transport of drugs. In sonophoresis, inertial cavitation potentially takes place in keratinocytes, lipid regions, and crevices of hair follicles, where the shock waves initiate structural disordering, forming diffusion channels to trigger drug delivery (Park et al., 2014). Although cavitation disrupts SC lipid structure and skin permeability, tissues located below are protected from damages, and the reduction in the skin barrier due to sonophoresis tends to be reversible (Rich et al., 2014). Since low-frequency ultrasound contains stronger inertial cavitation activities compared to high-frequency ultrasound, the former was found to be more efficient in topical drug delivery (Terahara et al., 2002; Zhang et al., 2017). In addition to inertial cavitation, temperature rise in skin by ultrasound may further potentiate drug permeability by affecting the liquidity of the phospholipid bilayer, leading to a direct change in membrane permeability (Daftardar et al., 2019) (O'Brien, 2007; Ueda et al., 1996). Compared to thermal effects, cavitation is considered to be the predominant mechanism responsible for drug delivery in sonophoresis (Park et al., 2014). With the variety of ultrasound parameters, systemic, regional, or programmed transdermal drug deliveries can be achieved by sonophoresis.

Practically, sonophoresis can be fulfilled by either simultaneous application of ultrasound and drugs or applying ultrasound in advance for a short period to permeabilize skin prior to drug administration. When applying a low-frequency ultrasound of 0.8 W/cm^2^ in combination with CsA, Liu et al. reported that almost sevenfold CsA was retained in the rat skin compared to simple passive diffusion, confirming the efficiency of sonophoresis in topical drug delivery (Liu et al., 2006). Using 20 kHz ultrasound 3 min in advance, Lifshiz et al. enabled the penetration of anti-psoriatic miRNA through the psoriatic SC barrier and into keratinocytes of immunodeficient mice transplanted with skin and psoriatic-activated peripheral blood mononuclear cells of human origin (Lifshiz Zimon et al., 2018).

Both being safe, noninvasive, transient, and painless, sonophoresis is superior to IP because not only charged but also uncharged drugs could be delivered. The major disadvantage of sonophoresis is the overpriced devices that are likely to be limited to hospital settings (Watkinson et al., 2016). Besides, considering the potential thermal and mechanical bioeffects of sonophoresis, it is imperative to take potential detrimental adverse reactions of sonophoresis into account. First, in inertial cavitation, the bubble may collapse rapidly and violently, leading to high localized tissue damage (Ahmadi et al., 2012; Miller et al., 2012). Then, owing to thermal alterations, sonophoresis may not be suitable for thermally unstable drugs.

### 3.2 Invasive Delivery

#### 3.2.1 Fractional Laser Ablation

Laser (Light Amplification by Stimulated Emission of Radiation) technology mediates transdermal drug delivery by directly changing the structure of skin and therefore promotes the percutaneous penetration and absorption of drugs. Due to risks of side effects, such as redness, swelling, and pain induced by conventional stripping lasers that work on the entire skin surface, the FLA procedure has been developed.

FLA works by fractionating laser beams of energy into a myriad of microbeams, which are capable of creating thousands of narrow and deep microchannels through heating skin tissues over 100°C, thus ablating tissues *via* vaporization of intracellular water. During FLA, the micro-thermal wounds produced by microbeams result in the formation of microscopic treatment zones (MTZs), which are composed of microchannels in skin as well as surrounding areas of thermally altered tissue, termed coagulation zones (CZs). Apart from MTZs and CZs, other adjacent tissues in FLA are kept free from damages. The highly condensed injuries within MTZs, typically ranging 50–250 μm in diameter, provide possibilities for keratinocytes from surrounding viable skin to migrate quickly towards MTZs, facilitating rapid re-epithelialization; hence, the micro-wound formed by FLA commonly recovers faster compared to conventional laser methods (Yu et al., 2018). The depth of individual microchannels varies from 25 to 1,000 μm, corresponding to the level of epidermis, or even dermis. Therefore, microchannels of FLA represent ideal pathways allowing skin permeation of macromolecular compounds (Fisher and Geronemus, 2005; Lapidoth et al., 2008). In addition, the surrounding CZs have been shown to be able to further promote topical uptake of drugs (Haak et al., 2017). Lasers involved in drug delivery include carbon dioxide (CO_2_) laser and erbium-doped yttrium aluminum garnet (Er:YAG) laser. Both CO_2_ and Er:YAG lasers are infrared lasers causing photothermolysis, but with distinctive penetration depth. Compared to CO_2_ laser, Er:YAG laser has a much higher absorption coefficient of water in skin, leading to less energy to ablate tissues and subsequently minimal thermal damages around the microchannels (Waibel et al., 2017). Since CO_2_ laser creates deeper MTZs, it is therefore preferable to Er:YAG laser for topical drug delivery, although both methods are effective at providing therapeutic agents through the SC (Searle et al., 2021).

Currently, laser-assisted drug delivery has been evaluated in various medical conditions, including psoriasis (Sklar et al., 2014). By using bovine hoof membrane as the *in vitro* model of human fingernail, Hiep et al. enhanced the *trans*-ungual delivery of MTX through disruption of nail barriers *via* FLA, providing possibilities for the treatment of nail psoriasis (Nguyen and Banga, 2018). In the improved delivery of macromolecules, Maria Lapteva et al. dedicated to verify the feasibility of topical delivery of a humanized anti-CD29 monoclonal antibody (OS2966) of 150 kDa using FLA. In psoriasis, CD29 integrin heterodimers mediate cell adhesion to a myriad of extracellular matrices, leading to dynamic tissue remodeling (Aljamal and Harrison, 2008; Rippa et al., 2013)*.* The topical application of its humanized antibody OS2966 could be of therapeutic significance in the treatment of psoriasis. In this study, based on an *in vitro* full-thickness skin model derived from the external side of the porcine ear, skin deposition and permeation of OS2966 increased almost linearly when skin was porated at a certain level of fluences. Additionally, visualization of Alexa 488-labeled OS2966 using confocal laser scanning microscopy (CLSM) confirmed that OS2966 was able to accumulate and then diffuse to the deeper dermis when pores created deeper than 120 μm. Hence, FLA would enable the delivery of macromolecules, like antibodies, which are beneficial for the treatment of localized moderate or severe psoriasis (Lapteva et al., 2019). Similarly, Río-Sancho et al. investigated the efficiency of Er:YAG FLA for topical cutaneous delivery of ETA, an anti-TNF-α medication of 150 kDa produced by fusing the TNF receptor to the constant end of the IgG1 antibody. In cutaneous biodistribution studies, the authors provided evidence that application of ETA-ablated skin enabled the delivery of therapeutically relevant amounts of ETA to the epidermis and upper dermis together with a low transdermal permeation, suggesting a minimal undesired systemic exposure (Del Río-Sancho et al., 2020). These findings are concordant with observed pharmacokinetics of transdermal drug delivery by FLA, reported by Laubach et al. After local administration of therapeutic compounds, drug molecules first spread along the vertical microchannels, and then expanded horizontally in the surrounding residual SC, where drugs accumulated. Afterwards, diffusion of drugs occurred at a deeper level, creating a drug concentration gradient. This long-term osmotic mechanism is supremely valuable in the treatment of moderate and severe localized psoriasis (Laubach et al., 2006).

In practical applications, considerations of factors affecting the delivery of drug by FLA should be taken into account, including parameters of applied laser, such as aperture and pulse number, the energy density of FLA, and the properties of drug (Ramez et al., 2018; Del Río-Sancho et al., 2020). In a preclinical study, FLA-assisted transdermal drug delivery was studied qualitatively and quantitatively by observing the effects of laser fluence, ablation depth, sample concentration, and novel topical formulations on the speed and deposition of drug delivery. By adjusting laser parameters, the drug delivery could be optimized to reach a desired depth, concentration, and diffusion rate according to specific clinical requirements (Del Río-Sancho et al., 2020). Moreover, the controlled delivery of OS2966 *via* FLA was shown dependent on laser parameters (i.e., aperture and pulse number) (Lapteva et al., 2019). Furthermore, Banzhaf et al. reported that with the increase of laser fluence, the opening time of the microchannel was prolonged, which provided a strategy for continuous transdermal penetration of drug molecules (Banzhaf et al., 2016). In addition, transdermal delivery of hydrophilic or low lipophilic drugs such as MTX and prednisone was closely related to the depth of microchannels, whereas lipophilic drugs such as lidocaine and IMQ showed weak association with the microchannels depth ablated by FLA (Chen et al., 2012; Taudorf, 2016).

As a physical penetration method, FLA-assisted transdermal drug delivery is advantageous due to less thermal injuries, the fact that it is controllable, the effective delivery of therapeutic agents, and the good compliance of patients. However, the majority of evaluations for FLA-mediated drug delivery was conducted with experimental animals, and relevant clinical evidence is eminently in demand. In particular, a comprehensive decision on clinically indispensable elements, such as laser parameters, drug properties, and treatment intervals, needs to be achieved before applying it as a therapy. In clinical psoriasis, FLA may work well on patients with mild-to-moderate cases, but it appears to be not practical for subjects with psoriasis on large areas of the body. Last but not the least, the development of smaller and more cost-effective laser equipment may further promote a broad application of FLA-assisted drug delivery.

#### 3.2.2 Microneedle

A microneedle device is a hybrid of the hypodermic needle and transdermal patch, employing hundreds of needles of micron size organized on a tiny patch in order to transport a sufficient number of drugs to a desired layer of skin. Upon application, microneedles rapidly create microscopic punctures across the SC layer into the underlying skin with minimal invasiveness (Waghule et al., 2019). Depending on the specific aim and function, different types of microneedles have been fabricated, including solid, coated, dissolving, hollow, and hydrogel microneedles. Solid microneedles are generally used prior to drug applications to primarily increase overall skin permeability. In coated microneedles, the drug-coating layer is designed to be located on the surface of individual needles, coming in direct and fast contact with skin. Dissolving microneedles are made of nontoxic polymers, which encapsulate drugs and eventually dissolve in skin. As for hollow microneedles, the empty cavity inside needles functions to provide space and protection for loaded drugs and enables passive or active injection of a liquified drug through hollow bores (Bhatnagar et al., 2017). Hydrogel microneedles are the novel forms of microneedles, consisting of swellable crosslinked hydrogels. Compared to other forms of previous versions, hydrogel microneedles inherit the hydrophilic nature of hydrogel, being ready to uptake water. In terms of transdermal drug delivery, therapeutic components could be directly incorporated into hydrogel microneedles during fabrication or loaded into a separate reservoir, which is attached to the top of hydrogel microneedles. The combination of microneedles with hydrogel aims to overcome the limitations of conventional hydrogel microneedles, hence providing higher drug-loading capacity, tunable drug release rate, and improved biocompatibility and biodegradability (Bhatnagar et al., 2019; Ahmed Saeed Al-Japairai et al., 2020; Turner et al., 2021).

Although applied in the form of numerous needles, microneedles are considered non-invasive and painless, as being microstructured means that they do not penetrate far enough into the skin to interact with and trigger pain receptors, which stay deeper in the dermis layer of the skin. Even though drugs are administered by needle injection, microneedles do not require specialized skills or personnel, as they are designed for self-administration to increase patient compliance, as well as single use to avoid cross-contamination of drugs (Shende et al., 2018; Xu et al., 2019; Jamaledin et al., 2020).

Practically, dissolvable microneedles are preferred due to better operability for patients. For instance, Du et al. developed a dissolving microneedle patch made of hyaluronic acid, which is excellent in water solubility, biocompatibility, biodegradability, and mechanical properties. By loading MTX, the resultant microneedles showed superior efficacies in alleviating the psoriasis-like skin inflammation compared to oral MTX administration at the same dosage (Du et al., 2019). Moreover, microneedles have been developed as an approach for simultaneous administration of multi-agents. For example, due to poor responses to topical or systemic glucocorticoid therapy as a result of glucocorticoid resistance, Wan et al. fabricated a dissolving microneedle patch containing sensitizers for glucocorticoid. Based on the fact that pyrin domain-containing 3 (NLRP3) is involved in increased glucocorticoid resistance, by directly and specifically disrupting NLRP3 *via* CRISPR-Cas9 within subcutaneous keratinocytes and immune cells, the dissolvable microneedle patch was designed to enable transdermal co-delivery of a combination of CRISPR-Cas9-based NLRP3 antagonists with glucocorticoids. In IMQ-induced psoriatic mouse models, continuous provision of the resultant microneedles for a week showed approximately 70% amelioration of the severity of psoriasis, as well as associated adverse effects (Wan et al., 2021). Likewise, in psoriatic arthritis, which is a type of inflammatory arthritis that occurs together with skin psoriasis, microneedle systems are favored for transdermal co-delivery of drugs in order to simultaneously alleviate psoriasis arthritis and psoriatic skin. In the layered dissolving microneedle designed by Yu et al., the topical therapeutic immunosuppressant, TAC, was loaded in the inter-layer and then transported within the skin of approximately 100 μm. Meanwhile, the widely used arthritis drug diclofenac sodium could be contrarily loaded into the tip layer of this microneedle and delivered up to 300 μm into the articular cavity (Yu K. et al., 2021).

Considering the microscale size of needles, breakage of microneedle tips may take place, which can be problematic if they lingered inside the skin. The possibility of skin irritation or allergy happening in sensitive skin induced by microneedles may exist, limiting the application of microneedles. To overcome these limitations, one of the main objectives of developing this technology is to select advanced materials, such as dissolvable polymers (Waghule et al., 2019).

## 4 Conclusion

To date, topical therapies, systemic treatment, and phototherapy remain the mainstay of clinical therapies for psoriasis. Though the advent of systemic administrations of biological agents has dramatically improved the clinical outcomes of moderate-to-severe psoriasis, a considerable medical need remains unmet. Accordingly, following the evolution of nanotechnology, a wide array of nanostructures has been engineered and employed as adjuvants of antipsoriatic strategies. With the view of providing more direct, effective, and rapid responses of antipsoriatic agents to local affected skin, the incorporation of nanotechnologies is extraordinarily favorable in topical drug delivery, as revealed in the following benefits. Firstly, incorporation of nanotechnologies dramatically enhances the efficiency of transdermal drug delivery, especially in psoriatic conditions characterized by highly packed SC, giving rise to increased drug availability in deeper skin layers and thus minimizing adverse effects *via* systemic applications. Secondly, the involvement of nanostructure expands the encapsulation capacity and efficacy of molecules with distinctive features, regardless of being hydrophilic or hydrophobic. Thirdly, an appropriate selection of nanomaterials offers protection for therapeutically active compounds and thus increases the stability of labile molecules. Moreover, the advantages of nanostructures as topical drug carriers have been reported to provide more intelligent assistance in controlling drug release as in micelles, strengthening bioadhesion of drugs by LCNPs, reinforcing anti-inflammatory effects in the case of MNPs, and improving skin hydration shown in NEs and nanogel. Furthermore, since the nanomaterials used for medical purposes are generally highly biocompatible, introduction of nano-substances for topical applications has been proven to be safe, and even less skin irritation, or allergy, or risk of damage has been observed in nanocarriers such as SLN and nanogel. In addition, as a promising remedy, the production of certain nanocarriers is scalable for industrial applications, with relatively simple, inexpensive, and environmentally friendly fabrication procedures.

With the increased diversity and availability of nanomaterials, selecting the most suitable nanostructure for the treatment of psoriasis has somehow become a puzzle. In the comprehensive understanding and analysis of benefits and limitations of the variety of nanocarriers that have been used in topical drug delivery in experimental psoriasis, it is noteworthy that pioneering studies have attempted to join different nanomaterials together to achieve a better therapeutic effect. Hence, it appears that a combination of more than two nanotechnologies depending on the physicochemical properties of loaded drugs and the conditions of psoriatic patients would constitute one of the prospects further putting forward the foreseeable clinical translation of nanomaterials in a topical drug delivery system for psoriasis. Meanwhile, current studies aiming to evaluate the efficacies of nanotech-assisted drug delivery are mostly based on an IMQ-induced psoriasis-like mouse model or normal human skin, which cannot represent the pathological alterations of human psoriatic skin. Great efforts based on investigations with more sophisticated experimental models, such as a 3D psoriasis skin model, are required before nanotechnologies are practically translated into clinical therapies.
